# Black rice diet alleviates colorectal cancer development through modulating tryptophan metabolism and activating AHR pathway

**DOI:** 10.1002/imt2.165

**Published:** 2024-01-15

**Authors:** Ling Wang, Yi‐Xuan Tu, Lu Chen, Ke‐Chun Yu, Hong‐Kai Wang, Shu‐Qiao Yang, Yuan Zhang, Shuai‐Jie Zhang, Shuo Song, Hong‐Li Xu, Zhu‐Cheng Yin, Ming‐Qian Feng, Jun‐Qiu Yue, Xiang‐Hong Huang, Tang Tang, Shao‐Zhong Wei, Xin‐Jun Liang, Zhen‐Xia Chen

**Affiliations:** ^1^ Hubei Hongshan Laboratory, Hubei Key Laboratory of Agricultural Bioinformatics, Hubei Key Laboratory of Metabolic Abnormalities and Vascular Aging, College of Life Science and Technology, College of Biomedicine and Health, Interdisciplinary Sciences Institute Huazhong Agricultural University Wuhan China; ^2^ Shenzhen Institute of Nutrition and Health Huazhong Agricultural University Shenzhen China; ^3^ Shenzhen Branch, Guangdong Laboratory for Lingnan Modern Agriculture, Genome Analysis Laboratory of the Ministry of Agriculture, Agricultural Genomics Institute at Shenzhen Chinese Academy of Agricultural Sciences Shenzhen China; ^4^ Department of Pharmaceutical Chemistry University of California‐San Francisco San Francisco California USA; ^5^ Department of Medical Oncology, Hubei Cancer Hospital, Tongji Medical College Huazhong Agricultural University Wuhan China; ^6^ Department of Pathology, Hubei Cancer Hospital, Tongji Medical College Huazhong University of Science and Technology Wuhan China; ^7^ Wuhan Myhalic Biotechnological Co., Ltd Wuhan China; ^8^ Wuhan Metware Biotechnology Co., Ltd Wuhan China; ^9^ Department of Gastrointestinal Oncology Surgery, Hubei Cancer Hospital, Tongji Medical College Huazhong Agricultural University Wuhan China

**Keywords:** black rice diet, colorectal cancer, gut metabolites, gut microbiome

## Abstract

Consumption of dietary fiber and anthocyanin has been linked to a lower incidence of colorectal cancer (CRC). This study scrutinizes the potential antitumorigenic attributes of a black rice diet (BRD), abundantly rich in dietary fiber and anthocyanin. Our results demonstrate notable antitumorigenic effects in mice on BRD, indicated by a reduction in both the size and number of intestinal tumors and a consequent extension in life span, compared to control diet‐fed counterparts. Furthermore, fecal transplants from BRD‐fed mice to germ‐free mice led to a decrease in colonic cell proliferation, coupled with maintained integrity of the intestinal barrier. The BRD was associated with significant shifts in gut microbiota composition, specifically an augmentation in probiotic strains *Bacteroides uniformis* and *Lactobacillus*. Noteworthy changes in gut metabolites were also documented, including the upregulation of indole‐3‐lactic acid and indole. These metabolites have been identified to stimulate the intestinal aryl hydrocarbon receptor pathway, inhibiting CRC cell proliferation and colorectal tumorigenesis. In summary, these findings propose that a BRD may modulate the progression of intestinal tumors by fostering protective gut microbiota and metabolite profiles. The study accentuates the potential health advantages of whole‐grain foods, emphasizing the potential utility of black rice in promoting health.

## Highlights

- The black rice diet (BRD) can slow down the development of colorectal tumors in *Apc* ^*Min/+*^ and azoxymethane/dextran sulfate sodium (AOM/DSS) colorectal cancer (CRC) model mice.
- The BRD increases the abundance of intestinal probiotics, such as *Bacteroides uniformis* and *Lactobacillus*, while reducing potential intestinal pathogens.
- The upregulated metabolites indole and indole‐3‐lactic acid in BRD are ligands of the aryl hydrocarbon receptor (AHR) pathway, which slow down the development of CRC by activating intestinal AHR receptor gene expression.
- A moderate consumption of BRD is expected to become a potential strategy for the prevention and treatment of CRC by improving gut microbiota and metabolites.

## Graphical abstract

Black rice diet can regulate gut microbial homeostasis in the colorectal cancer (CRC) mice model, enhance the abundance of beneficial bacteria, create aromatic hydrocarbon receptor ligand metabolites, activate the intestinal aryl hydrocarbon receptor (AHR) pathway, and suppress the development of CRC.

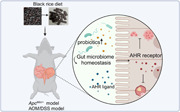

## INTRODUCTION

Globally, colorectal cancer (CRC) is the second most common cause of cancer‐related death and the third most common cancer [1]. As a gastrointestinal cancer, the risk of CRC is associated with a variety of diets [2, 3, 4, 5, 6]. The consumption of whole grains has been linked to a lower risk of CRC [7]. Recently, a meta‐analysis summarized the prospective evidence and reported a 10% decreased risk of CRC per additional 10 g/day total dietary fiber intake [8]. The potential protective impact of dietary fiber and consumption of whole grains on CRC risk is plausible from a biological perspective. Whole‐grain foods are key sources of dietary fiber and might mitigate the risk of CRC by promoting stool bulk, diluting harmful agents in the stool, and reducing transit time, ultimately limiting the interaction between carcinogens and the colorectal lining [9, 10, 11]. Moreover, the fermentation of fiber by bacteria generates short‐chain fatty acids, which could offer safeguards against CRC [12]. Antioxidants, vitamins, trace minerals, phytate, phenolic acids, lignans, and phytoestrogens are other ingredients found in whole grains that may also help prevent CRC [13]. Intake of whole grains has been linked to a lower risk of CRC due to their high folate and magnesium levels [14].

Unlike numerous other types of cancer, CRC actively interacts with trillions of gut microbes during the process of tumor development. In the meantime, the gut microbiota's makeup can either promote or inhibit the development of CRC [15]. Additionally, metabolites produced by microorganisms, such as bile acids and short‐chain fatty acids, might have a significant impact on the development of CRC [16, 17]. Although both whole grain consumption and a balanced gut microbiome are associated with an antagonistic effect on CRC development, the intricate relationship between whole grain intake, gut microbiome alterations, and metabolite changes in CRC development is not well understood.

Rice is a fundamental dietary staple in numerous regions globally. Black rice, being a type of rice, distinguishes itself from polished rice mainly due to its maintenance of the bran layer that is rich in various nutrients and bioactive compounds. Multiple studies have indicated that anthocyanin pigments in the bran layer of black rice exhibit significantly higher antioxidant activity [18, 19]. The bran layer also has a great fiber content. This fiber has the ability to attach to bile acids and carcinogens, which helps in the restoration of the lining of the colon [20, 21]. However, research on using black rice as a dietary system to explore its impact on the development of CRC is currently lacking. In this study, we used black rice diet (BRD), which is a whole‐grain diet rich in anthocyanin, to demonstrate the potential role of BRD in inhibiting CRC development using two CRC mouse models. We discovered that BRD antagonizes CRC development in mice through maintaining gut microbial homeostasis with enrichment of probiotic and depletion of pathogenic bacteria. This research is highly relevant for the advancement of preventative methods against cancer and the promotion of health‐oriented products, such as functional foods.

## RESULTS

### BRD protects against CRC development in both *Apc^Min/+^* and azoxymethane/dextran sulfate sodium (AOM/DSS) mouse models

BRD that meets nutritional requirements of adult mouse with a consistent concentration of energy and nutrients as a control diet (CD) is the prerequisite to study the health effects of black rice. We thus took AIN‐93M, the widely used purified diet made from refined ingredients for mature rodents [22], as the CD, while semipurified and isoenergetic diets containing 50% black rice, besides refined ingredients, as BRD.

**Figure 1:**
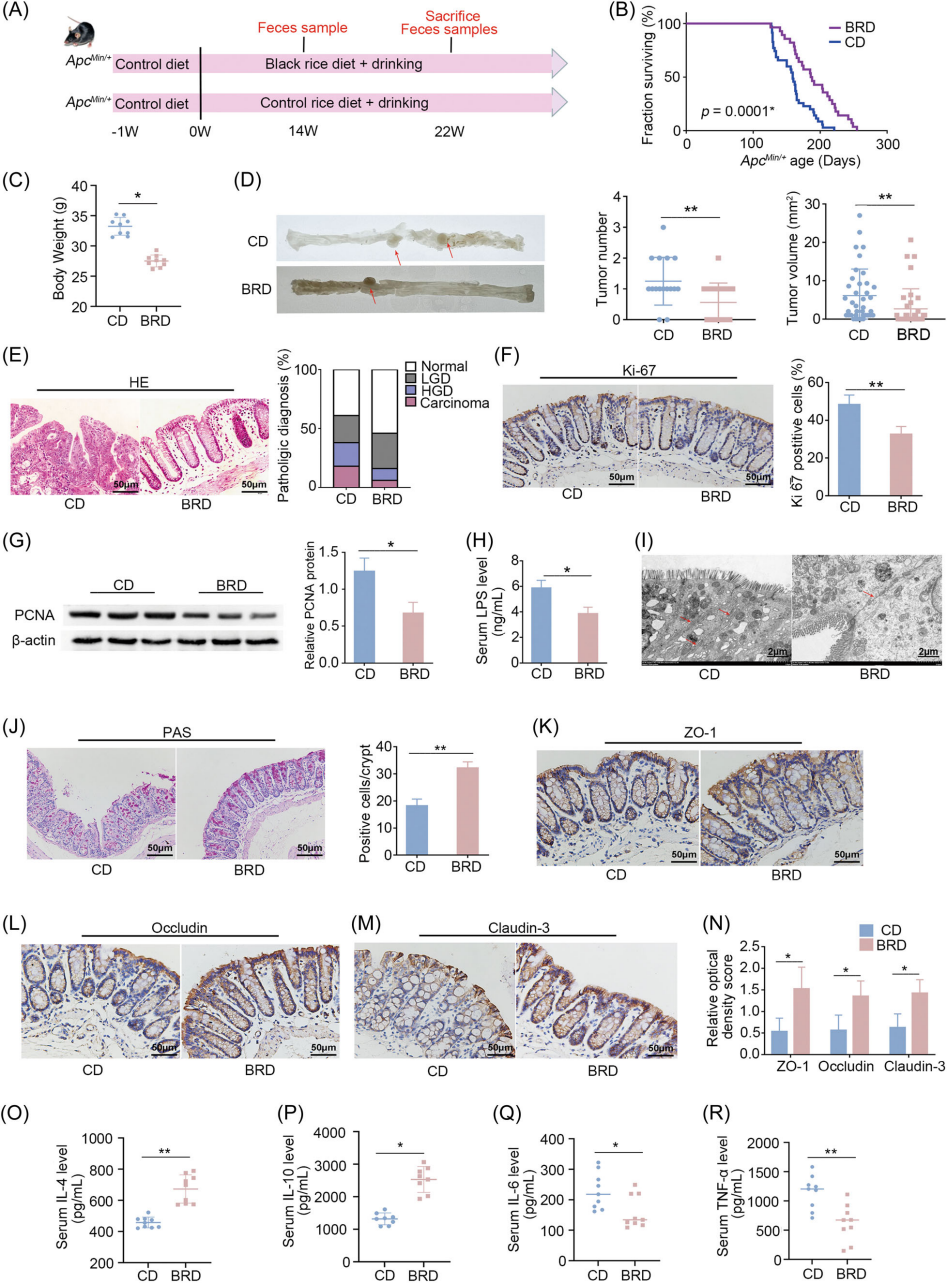
BRD against intestinal tumorigenesis in *Apc* ^*Min/+*^ mouse model. (A) Experimental design for *Apc* ^*Min/+*^ CRC mouse model and WT mice subjected to either a black rice‐fed diet or a control‐fed diet. (B) Enhanced survival observed in BRD‐fed mice (*n* = 28 per group) compared with CD‐fed mice (*n* = 35 per group). (C) Body weight of BRD‐fed and CD‐fed mice before killing (*n* = 9 per group). (D) Representative colon image at the time of killing. Tumor number and tumor volume in BRD‐fed and CD‐fed mice. The structures indicated by the red arrows are tumors. (E) H&E staining for pathological diagnosis of mice colons. Quantitative analysis of the pathological score employed the following criteria: 0, normal; 1, low‐grade dysplasia; 2, high‐grade dysplasia; and 3, carcinoma. (F) IHC staining for Ki‐67 in mice colons, accompanied by a quantitative analysis of the Ki‐67 index. (G) Expression levels of proliferating cell nuclear antigen (PCNA) protein in colon tissues of BRD‐fed and CD‐fed mice using western blot analysis with quantitative analysis. (H) Lipopolysaccharide (LPS) concentration in serum of BRD‐fed and CD‐fed mice in an *Apc* ^*Min/+*^ model. The relative protein levels are normalized to those of the control β‐actin. (I) Representative images of intercellular junctions captured by transmission electron microscopy. The structures highlighted by the red arrows are the focal points. (J) The number of colon goblet cells assessed by PAS staining. (K–N) IHC for the distribution of adhesion molecules ZO‐1, Claudin‐3, and occludin with quantitative analysis in colon tissues of BRD‐fed and CD‐fed mice. (O, P) Anti‐inflammatory interleukin (IL)‐4 and IL‐10 concentrations and (Q, R) pro‐inflammatory TNF‐α and IL‐6 concentrations in serum of BRD‐fed and CD‐fed mice in an *Apc* ^*Min/+*^ model. BRD, black rice diet; CD, control diet; CRC, colorectal cancer; H&E, hematoxylin and eosin; HGD, high‐grade dysplasia; IHC, immunochemistry; IL‐6, interleukin‐6; LGD, low‐grade dysplasia; PAS, periodic acid–Schiff; TNF‐α, tumor necrosis factor‐α; WT, wild type. *p < 0.05, **p < 0.01, N.S., no significant. Dot plots reflect data points from independent experiments.

To investigate the impact of black rice on CRC development, we administered CD or BRD to the gene mutant mouse model *Apc* ^*Min/+*^ from the completion of the adaptation feeding until natural death (Figure 1A). We observed a 15.4% extension in the maximum life span of mice fed BRD (255 days) compared with those fed CD (221 days), with an average life span extension of 20% in the BRD group (mean life span: 190.8 days) compared with the CD group (mean life span: 159 days) (Figure 1B). At harvest, BRD‐fed mice had lower body weight than CD‐fed mice (Figure 1C). Epidemiological data suggest that obesity is associated with a 30%–70% increased risk of colon cancer in men [23]. The colorectal tumor count and size were reduced in mice that were fed a diet containing BRD than in those that were given a CD, as shown in Figure 1D. Histological analysis of colon samples indicated that mice fed with BRD had a decreased incidence of adenocarcinoma, high‐grade dysplasia, and low‐grade dysplasia compared to CD‐fed mice (Figure 1E). The sections of the colon from animals fed with BRD showed a lower number of Ki‐67‐positive cells, indicating reduced cell proliferation (Figure 1F). Meanwhile, expressions of proliferating cell nuclear antigen (PCNA) were reduced in BRD‐fed mice (Figure 1G), indicating again decreased cell proliferation in BRD‐fed mice. The above findings indicated that BRD can alleviate the development of intestinal tumors in *Apc* ^*Min/+*^ mice.

The intestinal barrier serves as a protective mechanism that prevents the entry of harmful substances into the bloodstream, thereby averting a cascade of pathophysiological alterations [24]. To investigate the influence of gut barrier function on the inhibition of colorectal development by black rice, we assessed the effect of BRD on the permeability of the colon in mice. We measured the concentration of lipopolysaccharides (LPS), which are the main components of the outer membrane of Gram‐negative bacteria, in the serum. The serum content of LPS was lower in mice fed a BRD diet than in mice fed a CD diet (Figure 1H). The transmission electron microscopy analysis of the gut barrier structure confirmed that the colonic intercellular junctions in mice fed with BRD were relatively normal. However, mice fed with CD showed abnormalities in their colonic intercellular junctions, including widening of spaces in the apical junctional complex and paracellular gap (Figure 1I). Goblet cells are a type of specialized epithelial cells that play a crucial role in the creation of mucus barriers within the intestines [25]. Colon tissues were stained using periodic acid–Schiff, to determine the number of goblet cells in each crypt. The results showed that the average number of goblet cells was higher in mice fed with BRD compared with animals fed with CD (Figure 1J). Furthermore, the levels of tight junction proteins, which are essential for maintaining the integrity of the gut barrier, namely ZO‐1, occludin, and claudin‐3, were shown to be elevated in mice fed with BRD (Figure 1K–N). The results showed that BRD provided protection not only to the mechanical barriers of the gut but also to its chemical barriers.

Considering increased gut permeability can lead to chronic inflammation [26], we further examined the inflammation level of the CRC mice. The enzyme‐linked immunosorbent assay (ELISA) results demonstrated that the BRD downregulated the expression of pro‐inflammatory cytokines tumor necrosis factor‐α (TNF‐α) and interleukin‐6 (IL‐6), while upregulating anti‐inflammatory cytokines IL‐4 and IL‐10 (Figure 1O‐R). It suggested that the BRD lowered the serum inflammation levels in *Apc* ^*Min/+*^ mice.

To corroborate these findings, we employed the AOM/DSS‐treated C57BL/6 mouse model to ascertain whether the BRD's effect was specific to a particular CRC mouse model (Supporting Information S1: Figure S1A). Analogous to the *Apc* ^*Min/+*^ model, BRD‐fed AOM/DSS‐treated mice exhibited increased mean survival time of 29.5%, lower body weight, reduced tumor number and volume, improved gut barrier function, and attenuated inflammation levels (Supporting Information S1: Figure S1B–R). These consistent observations suggested that BRD may suppresses colorectal tumor development in multiple mouse models.

### Gut microbiota mediates the protective effects of BRD against CRC development

**Figure 2:**
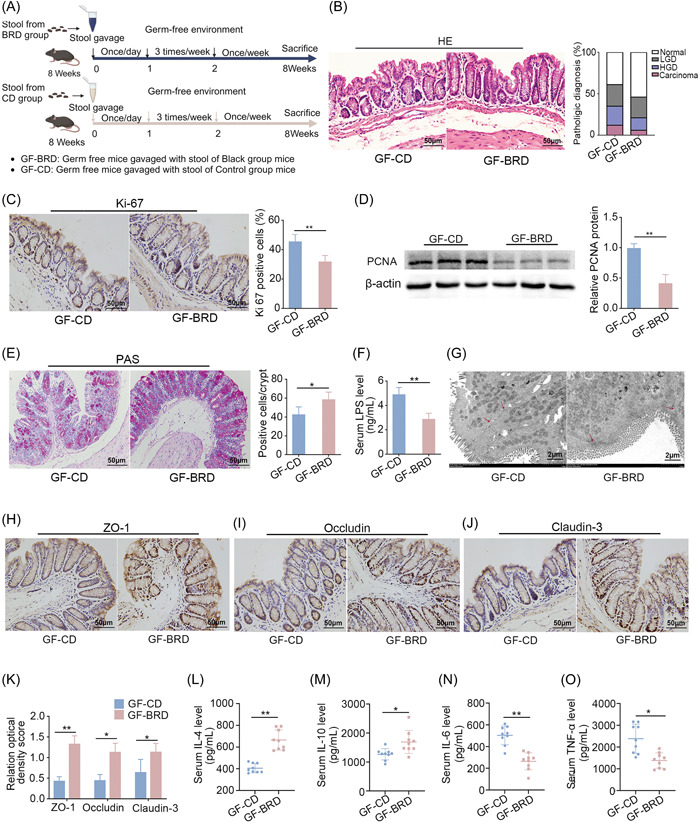
Black rice diet (BRD)‐modulated gut microbiota inhibits gut barrier dysfunction in germ‐free mice. (A) Experimental design for stools were transplanted from BRD‐fed mice and control diet‐fed mice to germ‐free mice (*n* = 7 per group) under CD. (B) H&E staining for pathological diagnosis of mice colons. Quantitative analysis of the pathological score employed the following criteria: 0, normal; 1, low‐grade dysplasia; 2, high‐grade dysplasia; and 3, carcinoma. (C) IHC staining for Ki‐67 and quantitative analysis of Ki‐67 index of GF‐CD and GF‐RBD mice colons. (D) Expression levels of cell proliferating indicating protein proliferating cell nuclear antigen (PCNA) in colon tissues of GF‐CD and GF‐BRD mice. The relative protein levels are normalized to those of the control β‐actin. (E) The number of colon goblet cells assessed by periodic acid–Schiff (PAS) staining. (F) Lipopolysaccharide (LPS) concentration in serum of GF‐CD and GF‐BRD mice. (G) Representative images of intercellular junctions captured by transmission electron microscopy. The structures highlighted by the red arrows are the focal points. (H–K) IHC for the distribution of adhesion molecules ZO‐1, claudin‐3, and occludin with quantitative analysis in colon tissues of GF‐CD and GF‐BRD mice. (L, M) Anti‐inflammatory IL‐4 and IL‐10 concentrations and (N, O) pro‐inflammatory tumor necrosis factor‐α (TNF‐α) and IL‐6 concentrations in serum of GF‐CD and GF‐BRD mice. GF‐BRD, germ‐free mice gavaged with fecal samples of BRD‐fed mice; GF‐CD, germ‐free mice gavaged with fecal samples of CD‐fed mice; H&E, hematoxylin and eosin; HGD, high‐grade dysplasia; IHC, immunochemistry; LGD, low‐grade dysplasia. *p < 0.05, **p < 0.01, N.S., no significant. Dot plots reflect data points from independent experiments.

Microbiota in the gut rely on dietary substrates and often serve as mediators for the cancer accelerating or suppressing effects of food [27, 28]. We thus checked the contribution of BRD‐modulated microbiota to intestinal health by transferring fecal samples of BRD‐fed mice or CD‐fed mice to germ‐free mice (Figure 2A).

To test the hypothesis, we examined cell proliferation, intestinal barrier function, and inflammation levels between GF‐BRD (germ‐free mice gavaged with fecal samples of BRD‐fed mice) and GF‐CD (germ‐free mice gavaged with fecal samples of CD‐fed mice) mice as we performed above in the comparison between BRD‐fed and CD‐fed CRC mice. In terms of cell proliferation, we found that GF‐BRD mice exhibited decreased high‐grade and low‐grade dysplasia compared with GF‐CD mice (Figure 2B). The attenuated Ki‐67‐positive cells in colon tissues were demonstrated in GF‐BRD mice compared with GF‐CD mice (Figure 2C). In keeping with this, PCNA protein was disintegrated in GF‐BRD mice (Figure 2D). In terms of gut barrier function, the decreased number of goblet cells in colon tissues (Figure 2E) and LPS level were demonstrated in serum of GF‐BRD mice (Figure 2F). Transmission electron microscopy confirmed the relatively normal colonic intercellular junctions in GF‐BRD mice, whereas confirmed the abnormalities of colonic intercellular junctions in GF‐CD mice, including widening of spaces in the apical junctional complex and paracellular gap (Figure 2G). Moreover, increased expression of ZO‐1, claudin‐3, and occludin were demonstrated in colon tissues of GF‐BRD mice compared with GF‐CD mice (Figure 2H–K). Consistently, ELISA results showed that the expression of anti‐inflammatory cytokine IL‐4 and IL‐10 was upregulated in GF‐BRD mice (Figure 2L,M), whereas expression of pro‐inflammatory cytokine TNF‐α and IL‐6 was downregulated in GF‐BRD mice (Figure 2N,O). All these results demonstrated gut microbiota and metabolite play a key role in mediating the protective effects of BRD in colorectal tumor development.

### BRD altered gut microbial composition and increased the abundance of *Bacteroides uniformis* in CRC mouse models

To further find out the beneficial microbiota against colorectal tumor development that could be upregulated by BRD, we sequenced and compared the metagenomes of BRD‐ and CD‐fed CRC mice, as well as CD‐fed wild‐type (WT) mice, at pre‐cancerous stage and tumor stage for both CRC models.

**Figure 3:**
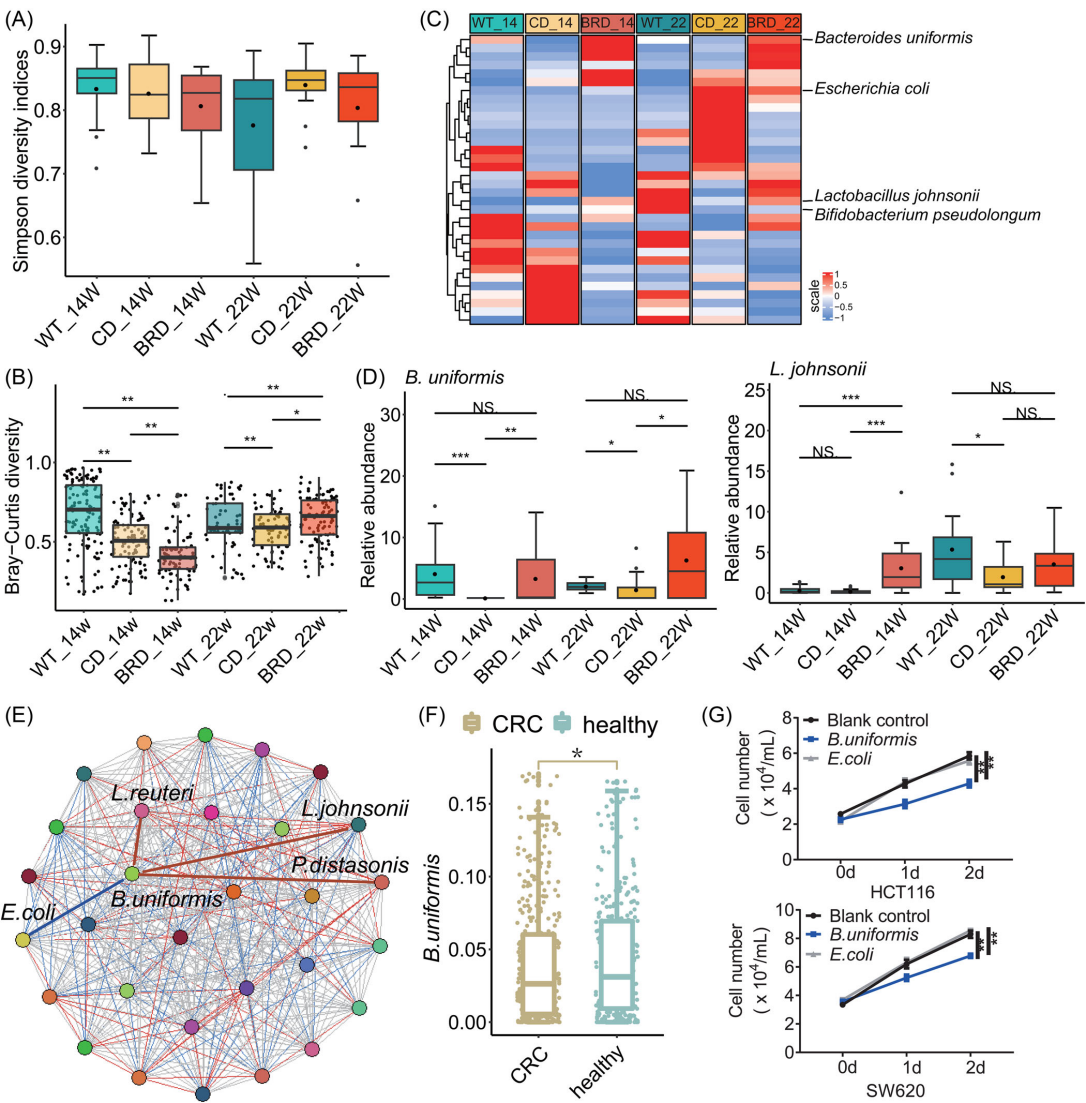
The black rice diet (BRD) altered gut microbial composition and increased the abundance of beneficial bacteria in the *Apc* ^*Min*/+^ model. (A) Alpha‐diversity analysis using the Simpson index in wild‐type mice, control diet (CD)‐fed, or BRD‐fed mice at 14 weeks (WT_14, *n* = 38 per group, CD_14, *n* = 15 per group, and BRD_14, *n* = 14 per group) and at 22 weeks (WT_22, *n* = 14 per group, CD_22, *n* = 22 per group, and BRD_22, *n* = 15 per group). (B) β‐diversity analysis using the Bray–Curtis distance. (C) Identification of marker microbes differentiating groups between black rice and CDs or between the wild type (WT) and control (*p* < 0.05, LDA > 2). (D) Relative abundance of *Lactobacillus johnsonii* and *Bacteroides uniformis*. (E) Co‐occurrence analysis: Spearman correlation coefficients between microbes. Different colors represent different bacteria. Red lines indicate positive correlations, blue lines indicate negative correlations, and gray lines indicate no correlation. (F) Relative abundance of *Bacteroides uniformis* in six cohort data sets via meta‐analysis. (G) Cell growth curves of CRC cell lines HCT116 and SW620 treated with *B. uniformis* and *E. coli* (as a negative control). BRD, black rice diet; CD, control diet; CRC, colorectal cancer; LDA, linear discriminant analysis. Data are expressed as mean ± SD. *p < 0.05, **p < 0.01, ***p < 0.001, N.S., no significant. Dot plots reflect data points from independent experiments.

In the *Apc* ^*Min/+*^ model, although the “Simpson Index” [29], used to measure species diversity in ecology, showed no differences among various dietary groups (Figure 3A), microbial composition variations were detected across different diet groups using the Bray–Curtis metric (Figure 3B), which is used to compare the similarity or dissimilarity between samples or communities [30]. To ascertain the specific bacterial changes associated with each diet group, we compared the BRD‐fed *Apc* ^*Min/+*^ mice and CD‐fed *Apc* ^*Min/+*^ mice, as well as the CD‐fed WT mice and CD‐fed *Apc* ^*Min/+*^ mice, at 14 weeks (represents the precancerous stage) and 22 weeks (represents the tumor stage). We observed that *B. uniformis* were more abundant in the BRD‐fed *Apc* ^*Min/+*^ mice and CD‐fed WT mice than CD‐fed *Apc* ^*Min/+*^ mice (Figure 3C,D and Supporting Information S2: Table S2). Co‐occurrence analysis revealed that *B. uniformis* was negatively correlated with harmful bacteria of *Escherichia coli* [31], while positively correlated with probiotics of *Lactobacillus johnsonii* [32] and *Lactobacillus reuteri* [33], suggesting *B. uniformis* may play a beneficial role in slowing down CRC development (Figure 3E).

We also compared the metagenomes of GF‐BRD and GF‐CD mice. Principal co‐ordinates analysis results showed that the β‐diversity of microbes of GF‐BRD mice and GF‐CD mice exhibited differences (Supporting Information S1: Figure S2A). GF‐BRD mice exhibited enrichment of *B. uniformis* (Supporting Information S1: Figure S2B and Supporting Information S2: Table S3), supporting the beneficial role of *B. uniformis* in CRC development.

In the AOM/DSS CRC model, the BRD group presented increased Shannon and Simpson indices (Supporting Information S1: Figure S3A), representing higher alpha diversity at both the pre‐cancerous stage (following the first DSS treatments) and tumor stage (following the third DSS treatments). Bray–Curtis distance analysis revealed differences between the BRD and CD (Supporting Information S1: Figure S3B), suggesting that the BRD enhanced gut microbial diversity and altered their composition. Similar to the *Apc* ^*Min/+*^ model, in tumor stage, *B. uniformis* was more abundant in CD‐fed WT mice and BRD‐fed CRC mice than CD‐fed CRC mice, while pathogenic *E. coli* was less abundant in CD‐fed WT mice and the BRD‐fed mice (Supporting Information S1: Figure S3C,D and Supporting Information S2: Table S4).

Additionally, co‐occurrence analysis demonstrated that *B. uniformis* was positively correlated with beneficial *L. johnsonii* and negatively correlated with harmful *E. coli* (Supporting Information S1: Figure S3E), suggesting again the advantageous effects of *B. uniformis* in CRC development.

### *B. uniformis* is a potential beneficial bacterium in patients with CRC

To explore whether *B. uniformis* also potentially plays a role in CRC development in humans, we analyzed six sets of published metagenome data to determine human intestinal bacterial abundance. Following a unified process, our meta‐analysis revealed that *B. uniformis* was more abundant in healthy individuals compared to patients with CRC (Figure 3F), suggesting the advantageous effects of *B. uniformis* in patients with CRC.

Additionally, we studied the effects of *B. uniformis* by coculture experiments with two distinct human CRC cell lines, SW620 and HCT116. Compared with cells cocultured with *E. coli* or blank control, cells cocultured with *B. uniformis* showed slower growth (Figure 3G), supporting again that *B. uniformis* may suppress CRC development in human.

### BRD altered intestinal metabolite composition and enhanced tryptophan metabolism pathway

To investigate the alterations in host and microbiota metabolism in response to different diet‐mediated microbial changes, we conducted fecal metabolic profiling of *Apc* ^*Min/+*^ and AOM/DSS models at the pre‐cancerous stage and tumor stage.

**Figure 4:**
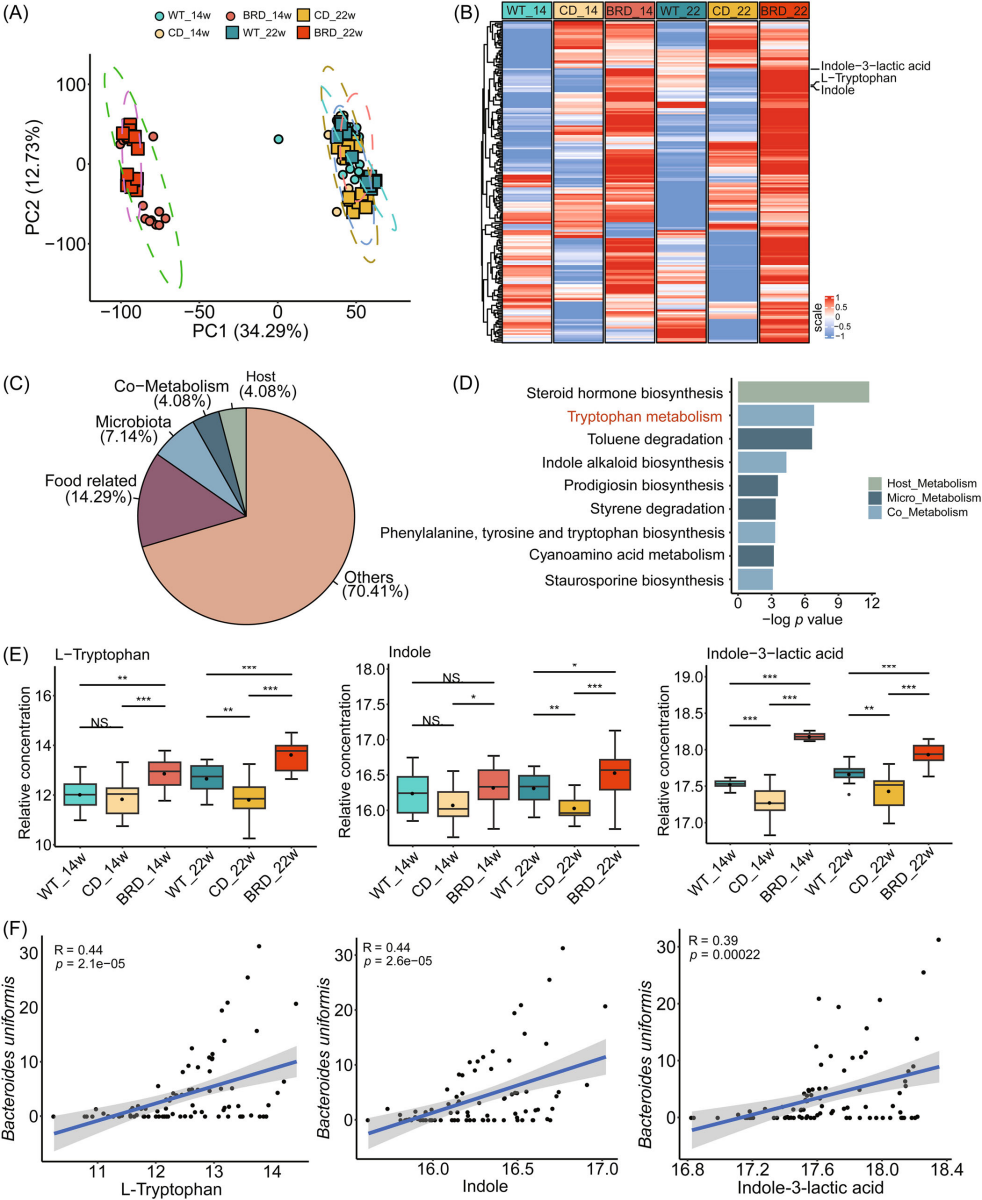
BRD altered intestinal feces metabolite composition and enhanced tryptophan metabolism pathway in the *Apc* ^*Min*/+^ model. (A) principal component analysis (PCA) plot for gut metabolomics analysis in wild‐type (WT), control diet (CD)‐fed, or BRD‐fed mice at 14 weeks (WT_14, *n* = 14 per group, CD_14, *n* = 16 per group, and BRD_14, *n* = 15 per group) and at 22 weeks (WT_22, *n* = 14 per group, CD_22, *n* = 16 per group, and BRD_22, *n* = 15 per group). (B) Identification of marker metabolites differentiating black rice and CDs or between WT and control. (C) Analysis of differential metabolite traceability: percentage of host (4.08%), microbial (7.14%), shared (4.08%), food‐related (14.29%), and other (70.41%) sources. (D) Enrichment analysis of the co‐upregulated metabolites from BRD‐fed *Apc* ^*Min*/+^ and CD‐fed WT mice. Tryptophan metabolic pathways are highlighted in red. (E) Three metabolites involved in the tryptophan metabolic pathway: tryptophan, indole, and indole‐3‐lactic acid. (F) Correlation analysis of *Lactobacillus johnsonii* and *Bacteroides uniformis* with indole and indole‐3‐lactic acid. Spearman correlation coefficient *R* and *p* values were marked. BRD, black rice diet; CD, control diet. Data are expressed as mean ± SD. *p < 0.05, **p < 0.01, ***p < 0.001, N.S., no significant. Dot plots reflect data points from independent experiments.

In *Apc* ^*Min/+*^ cancer model, principal component analysis (PCA) demonstrated that samples were clustered according to different diets, indicating that metabolite composition varied between BRD‐fed mice and CD‐fed mice (Figure 4A). To ascertain the differential metabolite associated with each diet group, we compared the BRD‐fed *Apc* ^*Min/+*^ mice and CD‐fed *Apc* ^*Min/+*^ mice, as well as the CD‐fed WT mice and CD‐fed *Apc* ^*Min/+*^ mice, at 14 weeks and 22 weeks. A total of 203 common differentially metabolites were identified between CD‐fed *Apc* ^*Min/+*^ mice and BRD‐fed *Apc* ^*Min/+*^ mice, as well as between CD‐fed WT mice and CD‐fed *Apc* ^*Min/+*^ mice (Figure 4B and Supporting Information S2: Table S5). Among them, indole and indole‐3‐lactic acid, were both among the top upregulated outlier metabolites in BRD‐fed mice at 14 weeks and 22 weeks (Figure 4B). Next, we classified the sources of these differentially metabolites. The results found that, in addition to other types, metabolites from microorganisms, hosts, and shared metabolites accounted for most of the remaining metabolites (15.30%) (Figure 4C). Enrichment analysis of co‐upregulated metabolites from the BRD‐fed *Apc* ^*Min*/+^ mice and CD‐fed WT mice revealed that the tryptophan metabolism pathway was enhanced (Figure 4D). l‐tryptophan, indole, and indole‐3 lactic acid within this pathway are considered metabolites of host‐microbial cross‐talk and are enriched under the influence of the BRD‐diet mice (Figure 4E). In addition, the spearman correlation coefficient showed that the three metabolites were positively correlated with *B. uniformis* (Figure 4F), which confirmed that all three could participate in the tryptophan metabolism pathway through the MetOrigin database (Supporting Information S2: Table S6).

To corroborate the generalizability of these findings, we assessed the metabolome of the AOM/DSS model in a similar manner. The results aligned with those from the *Apc* ^*Min/+*^ model, as PCA highlighted the substantial differences between the BRD and CD Supporting Information S1: Figure S4A). In total, 828 differential metabolites were identified during periods of inflammation and cancer between the BRD‐fed AOM/DSS mice and CD‐fed AOM/DSS mice, as well as between the CD‐fed WT mice and CD‐fed AOM/DSS mice (Supporting Information S1: Figure S4B and Supporting Information S2: Table S7). Among them, indole and indole‐3‐lactic acid were both one of the top upregulated outlier metabolites in BRD‐fed AOM/DSS mice (Supporting Information S1: Figure S4B). Notably, 24.95% of the differential metabolites originated from microbes, hosts, and both (Supporting Information S1: Figure S4C). In agreement with previous findings, these metabolites were also implicated in the tryptophan metabolic pathway (Supporting Information S1: Figure S4D).

To further elucidate the impact of the BRD on the host mice, serum metabolism was investigated. PCA results revealed that the metabolites in serum affected by the BRD‐fed *Apc* ^*Min/+*^ mice and the CD‐fed *Apc* ^*Min/+*^ mice displayed substantial differences at 22 weeks (Supporting Information S1: Figure S5A). A total of 223 metabolites exhibited significant disparities between the two groups (Supporting Information S1: Figure S5B and Supporting Information S2: Table S8), of which 31.82% originated from microorganisms and host‐microorganism sharing (Supporting Information S1: Figure S5C). Concentration analysis of the aforementioned serum metabolites demonstrated that tryptophan metabolism remained enriched in the BRD‐fed mice (Supporting Information S1: Figure S5D). These findings suggest that the BRD activates the tryptophan metabolism pathway involved in microbe–host cross‐talk, which may be one of the essential factors influencing the intestinal health of the host.

### Indole and indole‐3‐lactic acid inhibit cell proliferation and cell junction impairment

**Figure 5:**
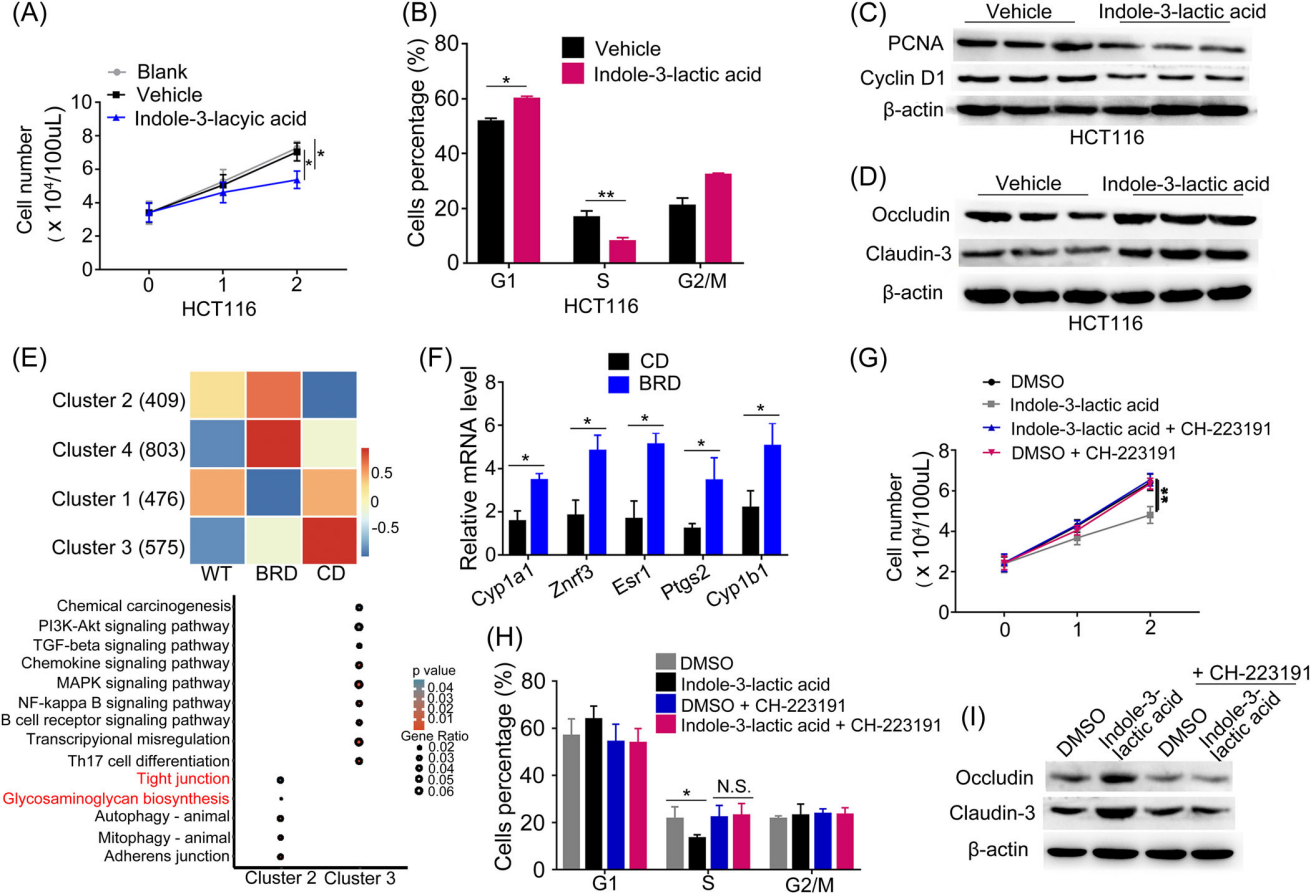
Indole‐3‐lactic acid activates host AHR to inhibit cell proliferation and cell junction impairment. (A) Cell growth curves of colorectal cancer (CRC) cell line HCT116 cells treated with indole‐3‐lactic acid and a vehicle (as negative control). (B) HCT116 cells treated with or without indole‐3‐lactic acid were stained with propidium iodide (PI) and analyzed using flow cytometry. (C) Expression levels of cell proliferation and cell cycle–associated proteins proliferating cell nuclear antigen (PCNA) and cyclin D1, in HCT116 cells treated with indole‐3‐lactic acid and a vehicle. (D) Expression levels of gut barrier function‐associated proteins occludin and claudin‐3 in HCT116 cells treated with or without indole‐3‐lactic acid. (E) The differentially expressed patterns of AHR core targets in colon tissues of wild‐type (WT), black rice diet (BRD)‐fed, and CD‐fed mice and KEGG pathways from core target enrichment in Cluster2 and Cluster3. (F) qRT‐PCR results showed that BRD promoted AHR downstream target gene expression. The relative RNA levels are normalized to those of the control *β‐actin*. (G) Cell growth curves of CRC cell line HCT116 colon cell lines treated with DMSO, indole‐3‐lactic acid, DMSO + CH‐223191 (a potent and specific antagonist of AHR), and indole‐3‐lactic acid + CH‐223191. (H) HCT116 cells treated with DMSO, indole‐3‐lactic acid, DMSO + CH‐223191, and indole‐3‐lactic acid + CH‐223191 were stained with PI and analyzed using flow cytometry. (I) Expression levels of gut barrier function‐associated proteins occludin and claudin‐3 in HCT116 cell lines treated with DMSO, indole‐3‐lactic acid, DMSO + CH‐223191, and indole‐3‐lactic acid + CH‐223191. The relative protein levels are normalized to those of the control β‐actin. AHR, aryl hydrocarbon receptor; DMSO, dimethyl sulfoxide; KEGG, kyoto encyclopedia of genes and genomes; qRT‐PCR, quantitative real‐time polymerase chain reaction. Data are expressed as mean ± SD. *p  < 0.05, **p  < 0.01, N.S., no significant.

To investigate the possible functional functions of metabolites altered by BRD in the development of CRC, two CRC cell lines were exposed to various metabolites. Levels of indole and indole‐3‐lactic acid were higher in mice fed with BRD than those in mice fed with CD. Coculture tests demonstrated that both indole and indole‐3‐lactic acid exerted inhibitory effects on cell growth in CRC cell lines HCT116 and SW620, as illustrated in Figure 5A and Supporting Information S1: Figure S6A–C, respectively. Meanwhile, cell cycle analysis showed that indole and indole‐3‐lactic acid treatment moderated cell cycle progression from G1 to S phase in HCT116 and SW620 cells compared with vehicle controls (Figure 5B and Supporting Information S1: Figure S6D–F). Consistent with these observations, downregulated protein expressions of PCNA and cyclin D1 were identified upon indole and indole‐3‐lactic acid treatment in CRC cell lines (Figure 5C and Supporting Information S1: Figure S6G–I). Furthermore, we examined whether indole and indole‐3‐lactic acid could affect epithelial barrier function. Increased protein expressions of occludin and claudin‐3 were observed in indole‐treated and Indole‐3‐lactic acid‐treated HCT116 and SW620 cells compared with their vehicle controls, inferring that indole and indole‐3‐lactic acid could protect barrier function (Figure 5D and Supporting Information S1: Figure S6J–L). The collective findings suggest that BRD hinders the progression of CRC, partially by increasing the levels of the anticancer metabolites indole and indole‐3‐lactic acid.

### Indole and indole‐3‐lactic acid activate host aryl hydrocarbon receptor (AHR) to inhibit CRC development

Indole, a metabolite derived from tryptophan metabolism by gut microbiota, has been shown to activate the AHR in the host [34]. AHR is a ligand‐activated transcription factor that regulates the expression of various genes involved in detoxification, cell proliferation, and immunity [35]. Activation of AHR by indole has been associated with inhibition of CRC development [36].

To obtain the core target genes of AHR and their functional information, 7497 target genes enriched in the vicinity of transcription start site (TSS) by 5000 bp upstream and downstream were downloaded from GTRD (http://gtrd.biouml.org/), and genes with site‐Count > 2 were considered as the core target genes of AHR, totaling 2805 genes (Supporting Information S1: Figure S7A). Subsequently, we explored the expression patterns of AHR downstream target genes. Using k‐means clustering method, we categorized these genes into four clusters based on expression patterns in three groups of intestinal samples (WT, CD, and BRD). Among these clusters, two interesting clusters of genes come into view. Cluster2 exhibited a pattern where gene expression was higher in the WT and BRD‐fed mice compared to the CD‐fed mice. In contrast, cluster3 showed a pattern where gene expression was lower in the WT and BRD‐fed mice (Figure 5E). After extracting genes from these two clusters, pathway enrichment analysis was performed. Genes in cluster2 were enriched in pathways closely related to gut function, including tight junctions and glycosaminoglycan biosynthesis. On the other hand, genes in cluster3 were predominantly enriched in pathways associated with immune and inflammation, such as immune cell differentiation and receptor signaling, nuclear factor‐κB signaling, and phosphatidylinositol 3‐kinase‐Akt signaling, indicating a close association between unregulated AHR target genes in response to the BRD and gut health.

To further validate whether the expression of downstream genes in the AHR signaling pathway is activated by indole and indole‐3‐lactic acid, this study conducted real‐time polymerase chain reaction to compare the gene expression levels between the CD‐fed and BRD‐fed mice. The results revealed upregulation of AHR downstream target genes, including *Cyp1a1*, *Cyp1b1*, *Znrf3*, *Esr1*, and *Ptgs2*, in the BRD‐fed mice (Figure 5F). Furthermore, in the colon tissues of germ‐free mice that received fecal transplants from mice in the BRD‐fed mice, we observed an elevated expression level of AHR downstream target genes *Znrf3* and *Cyp1b1* (Supporting Information S1: Figure S7B).

To further investigate whether indole and indole‐3‐lactic acid inhibit CRC development by activating the AHR signaling pathway, we selected the AHR pathway inhibitor CH‐223191 [37] to assess its effects on the proliferation of two CRC cell lines. Our findings revealed that the inhibitory properties of indole and indole‐3‐lactic acid on cellular proliferation were nullified upon the administration of CH‐223191 (Figure 5G and Supporting Information S1: Figure S8A). Additionally, the inhibitory effect of indole and Indole‐3‐lactic acid on cell cycle progression was eliminated upon the addition of inhibitors (Figure 5H and Supporting Information S1: Figure S8B), suggesting that indole inhibits the proliferation of CRC cells through the activation of the AHR pathway.

Furthermore, we investigated whether the inhibition of the AHR signaling pathway affects gut barrier function. We found that the expression of occludin and claudin‐3 proteins was increased in indole‐treated and indole‐3‐lactic acid‐treated HCT116 and SW620 cells (Figure 5I and Supporting Information S1: Figure S8C). Additionally, the protective effect of indole and indole‐3‐lactic acid on gut barrier function was eliminated upon the addition of inhibitors suggesting that indole and indole‐3‐lactic acid protect intestinal barrier function through the activation of the AHR pathway (Figure 5I and Supporting Information S1: Figure S8C). These findings provide additional evidence supporting the idea that BRD modulates gut microbiota in a manner that promotes the activation of the AHR signaling pathway, ultimately contributing to the attenuation of CRC development.

## DISCUSSION

**Figure 6:**
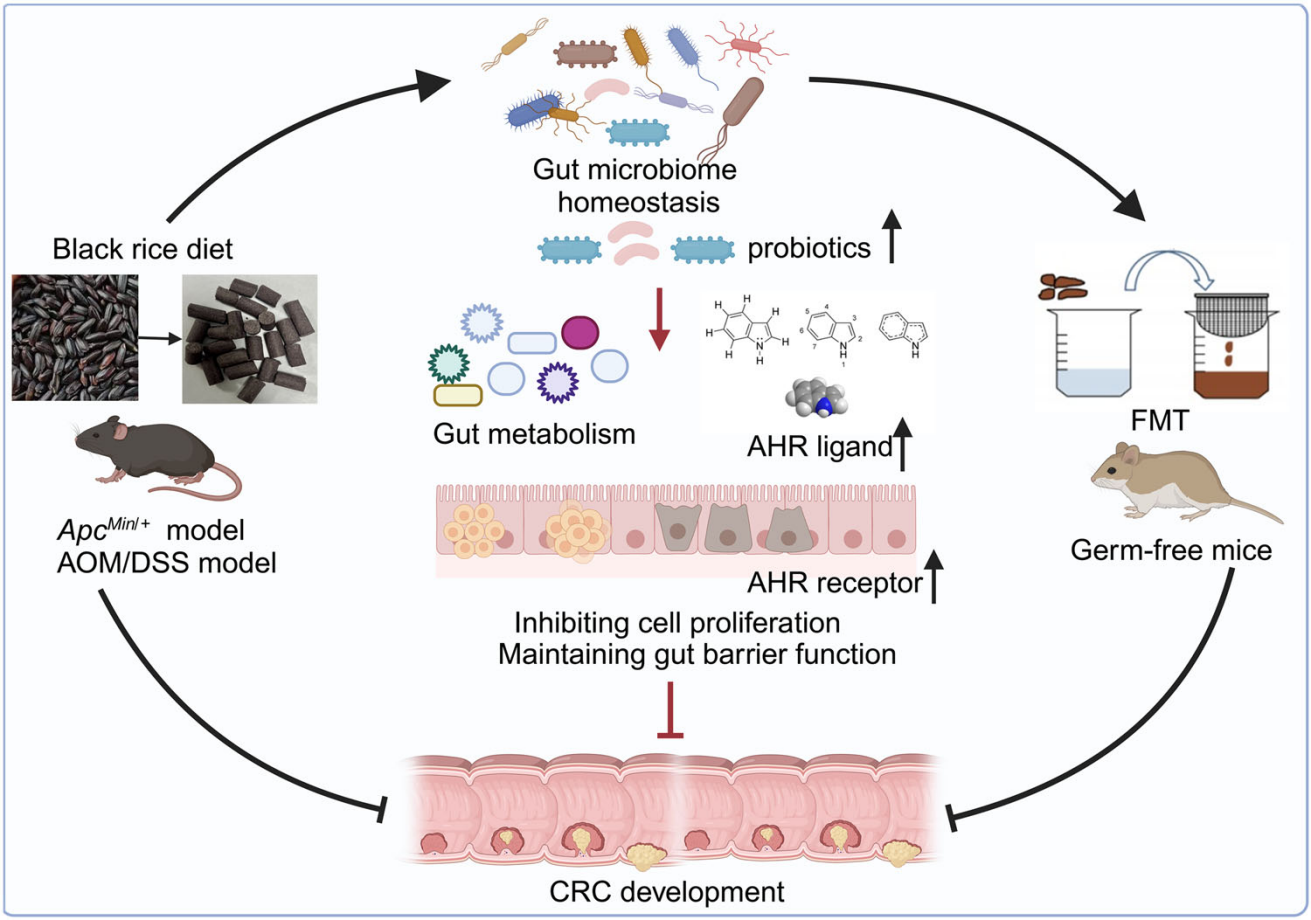
Black rice diet (BRD) alleviates colorectal cancer (CRC) development through modulating tryptophan metabolism and upregulating aryl hydrocarbon receptor (AHR) pathway. The BRD may attenuate CRC tumor development in CRC mouse models by promoting the abundance of protective gut microbiota and metabolites, as well as activating the host intestinal AHR pathway.

Gut microbiota disorder is one of the potential factors in the pathogenesis of CRC. Dysregulation of intestinal microbiota can increase intestinal mucosa permeability, aggravate intestinal mucosal epithelium damage, and ultimately lead to the deterioration of intestinal tumors [26, 38, 39]. Gut microbiota, shaped by lifestyle and nutritional habits, may play a key role in CRC pathogenesis [39]. The aim of this study was to investigate the effects of BRD on intestinal microbiota and metabolism in colon cancer mouse models and their adverse effects on the host, with the goal of regulating tumor development progression (Figure 6).

Black rice, a pigmented rice variant, is abundant in various nutritional and bioactive constituents. The components of this include necessary amino acids, effective fats, dietary fiber, a variety of vitamins and minerals, anthocyanins, phenolic compounds, γ‐oryzanols, tocopherols, tocotrienols, phytosterols, and phytic acid. These diverse constituents contribute to the health‐promoting attributes of black rice, rendering it a valuable dietary component for maintaining overall well‐being [40, 41]. Dietary fiber plays a role in the prevention and treatment of CRC. The consumption of whole grains was found to have a preventive effect, resulting in a modest decrease in the risk of developing CRC [7]. The main functions of dietary fiber are as follows: promotes bowel movement, beneficial gut flora balance, reduces cholesterol, and stabilizes blood sugar levels, antioxidant, and anti‐inflammatory effects [42, 43, 44]. Not only dietary fiber in black rice but also the abundant anthocyanins have a potential preventive and inhibitory effect on the development of CRC. The anthocyanins can inhibit the proliferation of colon cancer cells, protect the intestinal barrier function, and suppress the levels of inflammation in the serum, thus preventing and blocking the occurrence and development of CRC [45, 46].

In the present study, we observed that mice consuming a BRD displayed a reduction in body weight compared to control groups in both *Apc* ^*Min/+*^ and AOM/DSS CRC mouse models. Moreover, BRD‐fed mice exhibited a decreased number and size of intestinal adenomas, enhanced integrity of the gut barrier structure, ameliorated gut barrier function, diminished pro‐inflammatory cytokine levels, and elevated anti‐inflammatory cytokine levels. In addition, findings from fecal microbiota transplantation experiments demonstrate that germ‐free mice receiving fecal microbiota from the BRD group had a more complete intestinal barrier function when compared to germ‐free mice receiving the CD group mice. These results suggest that BRD can regulate gut microbial homeostasis to some extent and potentially mitigate the progression of CRC tumors in CRC mouse models.

Next, we revealed that BRD‐fed mice and CD‐fed mice exhibit distinct microbiota composition. We found that BRD increased the number of beneficial gut bacteria in both models, including *B. uniformis* and *Lactobacillus*. Studies have demonstrated its ability to reduce high‐fat diet‐induced metabolic changes, improve dendritic cell antigen presentation, and affect the proliferation of CD cells in obesity models, as well as reduce inflammation [47, 48, 49, 50]. Moreover, it has been reported to be more abundant in the feces of healthy volunteers than patients with CRC and increased in a fiber‐rich environment [47, 51]. By analyzing six public data sets, we also verified the conservation of this bacterium in human and mouse guts and its greater enrichment in healthy volunteers. To demonstrate a causal relationship between pathogenic microbiota and disease phenotype, we further evaluated the effects of BRD‐ and CD‐mediated intestinal microbiota on germ‐free mouse guts. The results showed that BRD‐mediated intestinal microbiota induced a less severe degree of intestinal cancer and a more intact intestinal barrier function. Notably, *B. uniformis* remained more abundant in the black rice fecal microbiota transplantation group. Anthocyanins and anthocyanin monomers derived from black rice have demonstrated prebiotic activity [52, 53]. To further investigate the potential reasons for the enrichment of *B. uniformis* in the BRD group, we first performed targeted anthocyanin sequencing to examine the black rice extract and diet. The results showed that cyanidin‐3‐glucoside was the most abundant anthocyanin component in both the black rice extract and diet. Addition of an appropriate amount of C3G to the *B. uniformis* culture medium promoted its growth. These findings suggest that C3G may be a key factor contributing to the enrichment of *B. uniformis* in the BRD group. Furthermore, both cancer cell lines SW620 and HCT116 exhibited suppressed growth under *B. uniformis* treatment. These results suggest that black rice inhibits colon cancer development by promoting *B. uniformis* proliferation.

*Lactobacillus gallinarum* produces and breaks down l‐tryptophan, releasing ILA, to fight CRC [54]. In our study, the tryptophan metabolic pathway was enriched in healthy, BRD, and WT groups, and related metabolites such as tryptophan, indole, and indole‐3‐lactic acid were utilized and produced by both *Lactobacillus* and *B. uniformis*. Indole and indole‐3‐lactic acid are compounds that naturally exist in foods such as vegetables, fruits, grains, and meats and have certain anticancer effects [54, 55]. Indole activates the Nrf2 signaling pathway to promote the self‐repair ability of intestinal mucosal cells and reduce the damage of harmful substances to the intestines [56]. At the same time, indole can also inhibit the Wnt signaling pathway to suppress the growth of intestinal tumors [57]. Indole‐3‐lactic acid also has an anti‐CRC effect, mainly by activating the AHR signaling pathway. Indole‐3‐lactic acid can bind to AHR receptors, thereby activating the AHR signaling pathway, inhibiting the growth and spread of CRC cells, and reducing the incidence of CRC [58]. Moreover, indole and indole‐3‐lactic acid inhibited cancer cell growth in our study. These findings suggest that *B. uniformis* and *Lactobacillus* may play a synergistic beneficial role through their metabolites in a BRD.

Furthermore, we investigated the mechanisms through which indole and indole‐3‐lactic acid affect the host. The relationship between the AHR pathway and the development of CRC has been widely studied [36, 59]. AHR activation has been shown to have both protumorigenic and anti‐tumorigenic effects, depending on the context and stage of cancer development. AHR activation can promote tumor growth by inducing the expression of genes involved in cell proliferation and survival, angiogenesis, and inflammation. On the other hand, AHR activation can also have anti‐tumorigenic effects by promoting the differentiation and activation of immune cells that can target and eliminate cancer cells [60]. In this study, we found that AHR and its downstream target genes were upregulated in the BRD‐fed mice at the intestinal gene transcription level. The inhibitory effect of indole disappeared with the addition of AHR pathway inhibitors, indicating that black rice‐enriched indole and indole‐3‐lactic acid acted as an AHR ligand to activate the AHR pathway, thus alleviating CRC development.

## CONCLUSION

In conclusion, our study uncovers that BRD suppressed colorectal development in both *Apc* ^*Min/+*^ and AOM/DSS mice when compared to CD‐fed mice, and it prolonged the life span of CRC model mice. Our findings suggest that BRD may protect against intestinal development by promoting a protective gut microbiome and metabolite profile, which can counteract gut microbial dysbiosis and safeguard gut barrier function in CRC model mice. The results also suggest the great potential value of whole‐grain staple food, especially the black rice staple food, in human health.

## METHODS

### The BRD

According to the AIN‐93M diets [61], a formula for purified diet used for mice was designed. Firstly, the composition of the rice produced in the current year was determined to ascertain the content of protein, calcium, phosphorus, and other components. Subsequently, the formula was balanced using reference ingredients provided by the AIN‐93M diets. The diet was manufactured by Readydietech Co., Ltd., Shenzhen, China. The rice was first mixed with drikold and then ground into powder at 4°C. It was then uniformly mixed with ingredients such as casein and subjected to extrusion and granulation at room temperature. After granulation, the diet was dried at 32°C for 18 h to ensure presence of 12% moisture content, followed by vacuum packaging and irradiation sterilization and storage at −20°C. The rice used for diet production is produced annually, the ingredients are provided by the company, and diet production is conducted every 3 months to ensure freshness and quality. The detailed feed nutrient formula is displayed in Supporting Information S2: Table S1.

### Conventional CRC mouse models

In the AOM/DSS model, 4‐week‐old C57BL/6 mice (Hunan SJA Laboratory Animal Co., Ltd) were acquired and subjected to a 1‐week adaptive feeding period before being randomly divided into two groups based on their body weight, receiving either a BRD (Readydietech Co., Ltd) (45 mice per group) or a CD (45 mice per group). The mice were given an intraperitoneal injection of 10 mg/kg AOM (Merck) at 8 weeks of age. This was followed by three cycles of DSS (MP Biomedicals) administration to model colitis‐associated CRC [62]. Each cycle consisted of 7 days with 2.0% DSS‐supplemented drinking water, followed by 14 days of regular water. The *Apc* ^*Min/+*^ mice were provided by a national rodent seed center (SJA Laboratory Animal Co., Ltd). AOM/DSS mice used for phenotypic experiments were euthanized at day 156, and *Apc* ^*Min/+*^ models were killed at Day 154 under anesthesia to collect serum and various tissue samples from each group. According to European Directive 2010/63, animals of the same sex (four mice per cage) should be housed in pairs during testing to ensure their social needs are met. The spatial arrangement of the mice within the same group was randomized. The mice were kept in a controlled environment with a specific‐pathogen‐free condition and a 12 h cycle of light and darkness. All procedures complied with the guidelines approved by the Animal Experimentation Ethics Committee of Huazhong Agricultural University.

### Life span measurement

The *Apc* ^*Min/+*^ mice used for survival rate statistics were randomly divided into a BRD group (*n* = 35) and a control group (*n* = 37). After a 2‐week adaptation feeding period, they were respectively fed with black rice feed and control group feed. During the life span statistics, we only recorded the life spans of *Apc* ^*Min/+*^ mice that developed CRC (the mice in each group were used to determine survival rates until 34 weeks of age) [63]. Autopsies were performed on the intestines to verify the existence of tumors. Mice were killed if they met any of the following criteria: (1) inability to feed or drink, (2) bleeding from a tumor or other ailment, or (3) being laterally recumbent, meaning they did not respond to stimulation or were unable to regain an upright position [64].

### Germ‐free mouse models

To investigate the direct impact of gut microbiota modulated by a BRD on healthy colonic mucosa, germ‐free BALB/c mice (Gempharmatech Co., Ltd) at 8 weeks of age were divided into two groups (seven mice per group) and maintained on a CD. The mice were then gavaged with fecal samples obtained from either BRD‐fed or CD‐fed mice. In summary, 1 g of stool samples was thoroughly mixed in 5 mL of phosphate‐buffered saline (PBS). The recipient mice were subsequently transplanted with 200 μL of the solution utilizing gastric gavage. In the first week, fecal microbiota transplantation is conducted daily. In the second week, it is done three times a week, and in the subsequent weeks, it is administered once a week. Animals were killed at 8 weeks postgavage.

### Shotgun metagenomics sequencing and analysis

After conducting Sample Quality Control, 500 nanograms of meta‐DNA was fragmented using ultrasound using a Covaris E220 instrument from Covaris, located in Brighton, UK. The fragments were then selected to be within the size range of 300–700 bp using magnetic bead size selection. The DNA segments underwent repair and were then joined together with indexed adaptors. The ligation product was subjected to PCR amplification, followed by exon probe hybridization and streptavidin bead capture. The DNA that was captured was amplified using PCR and then transformed into a single‐stranded circular (ssCir) library. The ssCir library underwent rolling circle amplification to generate DNA nanoballs (DNBs). These DNBs were then loaded onto a flow cell and sequenced using the DNBSEQ Platform.

The fecal metagenomic shotgun sequences underwent quality filtering using the “—trimmomatic‐options” in Kneaddata (v0.10.0). Reads that were <50 nucleotides were excluded. Reads that had been filtered were aligned to the mouse genome (C57BL) using bowtie2 software and any mouse DNA present in the reads was subsequently eliminated. MetaPhlAn3 software, version 3.0.14, was utilized to measure the taxonomic makeup of microbial communities in all metagenomic samples. Additionally, HUMANn3 software version 3.0.1 was employed to examine the abundance of pathways and gene families [65]. LEfSe [66] was utilized to screen differential bacteria, considering candidates meeting the criteria with linear discriminant analysis > 2 and *p* < 0.05.

### Metabolomics analyses and metabolite profiling

The ultra‐performance liquid chromatography‐mass spectrometry raw data were transformed into mzXML format using the MSConvert tool (http://proteowizard.sourceforge.net/downloads.shtml) to facilitate subsequent analysis. Nonlinear retention time correction, peak filtration, and extraction were performed using the XCMS package in R (v3.4.1). The profile obtained, which includes the mass‐to‐charge ratio (m/z), retention time, and ion intensity, was subjected to additional analysis using the metaX package in R (v3.4.1). This analysis involved signal correction and peak normalization, which were carried out using quality control samples as a basis.

The missing value in the data was replaced with the smallest value, and then, only the metabolites with a quality control variance less than 0.2 were kept. The metabolomics data underwent logarithmic processing to approach a normal distribution. A t‐test was then employed to identify potential candidates, with the false discovery rate (FDR) *p* value to be less than 0.05 serving as one of the screenings criteria. The additional requirement was that the variable importance in projection score be >1, as determined by the orthogonal partial least‐squares discrimination analysis using the R package ropls (version 1.20.0) after standardized data [67]. The Spearman correlation was used to calculate the associations between different bacteria and metabolites. The ComplexHeatmap (v2.4.3) package was used to construct the heatmap R package [68]. Metabolite source categorization and metabolite enrichment study were conducted utilizing the MetOrigin database [69]. And the functional items of Deep MetOrigin Analysis confirmed the real association between metabolites and microorganisms and the network construction.

### Public cohort metagenomics analysis

Public data for six colon cancer metagenomic sets [70, 71, 72, 73, 74] were downloaded from the curatedMetagenomicData R package [75] and samples for other diseases such as adenomas, type 2 diabetes, fatty liver, and hypertension were excluded from the available data sets. To ensure consistency and data quality, the above standard sample data were then rigorously filtered: (1) samples with low relative readings (≤1,000,000) were subsequently excluded, possibly due to low sequencing depth and contamination of host readings. (2) Removal of outliers and suspected contamination cases, including high species counts (species readings ≥ 50% of the total) and low species counts (species readings ≤ 0.01% * 1/*n*; *N* is the number of samples for different disease states in each study). (3) Species with low abundance are discarded (species read count ≤ 0.001 of total number). Public data metagenomic analysis adopts the meta‐analysis method, and the method of identifying major confounding factors is consistent with previous studies [76].

### Indole and indole‐3‐lactic acid treatment, cell proliferation, and cell cycle analysis

Indole (catalog number HY‐W001132, MedChemExpressA) and indole‐3‐lactic acid (catalog number HY‐113099, MedChemExpress) were procured from MedChemExpress. CRC cell lines HCT116 and SW620 were seeded in 96‐well plates at 5000 cells/well. Cells were treated with PBS, or vehicle, or 2 μmol/L Indole, or 2 μmol/L Indole‐3‐lactic acid in Dulbecco's modified Eagle medium (DMEM) supplemented with 10% fetal bovine serum for up to 2 days. For cell counting, cells were trypsinzed and counted daily.

For the analysis of the cell cycle, the cells were deprived of serum for 24 h and kept in DMEM supplemented with either 0.1% fatty acid‐free bovine serum albumin/vehicle or 2 μmol/L indole, or 2 μmol/L indole‐3‐lactic acid in 0.1% fatty acid‐free bovine serum albumin for 12 h. The cells were treated with 70% ethanol to immobilize them, then stained with propidium iodide, and finally examined using flow cytometry.

### RNA sequencing and data analysis

FastQC (https://www.bioinformatics.babraham.ac.uk/projects/fastqc/) was used to check the quality of the raw sequence fastq files. High‐quality pruning was performed using fastp (v0.23.2) [77]. Paired end reads were mapped to the reference genome of Ensembl (GRCm39) using HISAT2 (v2.2.1) [78]. SAMtools (v1.14) [79] is used to sort and index align BAM files. Reads were counted using the featureCounts program in the Subread (v2.0.1) [80] package. DESeq. 2 (v1.28.1) [81] was used to screen different groups of differential genes, while ClusterProfiler (v3.16.1) [82] was used for enrichment analysis.

### Statistical analysis

Statistical analysis was performed using GraphPad Prism 8.0 (GraphPad Software Inc.) and R software, version 4.0.3. The comparison of categorical variables was performed by the *χ* ^2^ test. Nonparametric Kruskal–Wallis tests were used to compare microbial differences between the three groups (WT, CD, and BRD groups). Standardized (Z‐score) data were used for metabolite analysis. The covariate effect of species diversity was examined by the multiple linear regression method. An intergroup difference test (permutational multivariate analysis of variance) was performed for the Euclidean distance of metabolites and the Bray–Curtis distance of bacteria. *p* < 0.05 and Benjamini–Hochberg corrected for FDR values were used for statistical significance. Spearman's rank correlation coefficient was used to estimate microbe‐microbe or microbe‐metabolite correlation.

Additional methods are provided in the Supporting Information S1.

## AUTHOR CONTRIBUTIONS

Ling Wang, Yi‐Xuan Tu, Lu Chen, Shao‐Zhong Wei, Xin‐Jun Liang, and Zhen‐Xia Chen conceived and designed the research. Ling Wang, Yi‐Xuan Tu, Yuan Zhang, Lu Chen, Shu‐Qiao Yang, Shuai‐Jie Zhang, Ke‐Chun, Yu, Shuo Song, Hong‐Li Xu, Zhu‐Cheng Yin, and Tang Tang performed experiments. Ling Wang, Yi‐Xuan Tu, and Yuan Zhang analyzed the data. Hong‐Kai Wang provided the mouse diets. Ling Wang, Yi‐Xuan Tu, and Zhen‐Xia Chen wrote the manuscript. All authors have read the final manuscript and approved it for publication.

## CONFLICT OF INTEREST STATEMENT

The authors have declared no competing interests.

## ETHICS STATEMENT

The ethics application (No. HZAUMO‐2022‐0146) was approved by the Animal Experimentation Ethics Committee of Huazhong Agricultural University.

## Supporting information


**Figure S1**: Black rice diet against intestinal tumorigenesis in AOM/DSS mouse model.
**Figure S2**: Germ‐free mice receiving fecal microbiota transplantation exhibit altered gut microbial composition.
**Figure S3**: The black rice diet altered gut microbial composition and increased the abundance of beneficial bacteria in AOM/DSS model.
**Figure S4**: Black rice diet altered intestinal feces metabolite composition and enhanced tryptophan metabolism pathway in AOM/DSS.
**Figure S5**: Black rice diet altered intestinal serum metabolite composition and enhanced tryptophan metabolism pathway in *Apc^Min^* ^/+^ mice.
**Figure S6**: Indole and Indole‐3‐lactic acid inhibit cell proliferation and cell junction impairment.
**Figure S7**: The AHR pathway in the gut of germ‐free mice receiving black rice fecal microbiota transplantation was activated.
**Figure S8**: Indole activates host AHR to inhibit colorectal cancer development.

**Table S1**: Diet composition.
**Table S2**: The differential microbes of *Apc^Min^* ^/+^ in BRD and CD.
**Table S3**: The differential microbes of GF‐CD and GF‐BRD.
**Table S4**: The differential microbes of AOM/DSS in BRD and CD.
**Table S5**: Upregulated common metabolites in *Apc^Min^* ^/+^ mice on BRD and WT mice on CD.
**Table S6**: Information of participation of *Lactobacillus johnsonii* and *Bacteroides uniformis* in the tryptophan metabolism pathway through the MetOrigin database.
**Table S7**: Upregulated common metabolites in AOM/DSS mice on BRD and WT mice on CD.
**Table S8**: Differential metabolites in BRD and CD in *Apc^Min^* ^/+^ mice serum.

## Data Availability

The data sets generated in the current study are available in the National Genomics Data Center, Beijing Institute of Genomics, Chinese Academy of Sciences/China National Center for Bioinformation (GSA: CRA008369 and OMIX: OMIX001920) and are publicly accessible at https://bigd.big.ac.cn/. Code and all analysis results can be found at https://github.com/Yichel518/Dietary-analysis-for-CRC. Supplementary materials (methods, figures, tables, scripts, graphical abstract, slides, videos, Chinese translated version, and update materials) may be found in the online DOI or iMeta Science http://www.imeta.science/.
