# Correction to “Black rice diet alleviates colorectal cancer development through modulating tryptophan metabolism and activating AHR pathway”

**DOI:** 10.1002/imt2.70039

**Published:** 2025-04-29

Wang, Ling, Yi‐Xuan Tu, Lu Chen, Ke‐Chun Yu, Hong‐Kai Wang, Shu‐Qiao Yang, Yuan Zhang, et al. 2024. “Black Rice Diet Alleviates Colorectal Cancer Development Through Modulating Tryptophan Metabolism and Activating AHR Pathway.” *iMeta 3*(1): e165. https://doi.org/10.1002/imt2.165

In Wang et al. [1], the content of C3G in Table S1 of Supporting Information should be corrected; the content of C3G (accounted for 6.59 μg/mg) should be 0.659 μg/mg.

The correct table should be as follows:

**Table:** 

Percentage (%)	Control diet	Black rice‐diet
Protein	13	12
Carbohydrate	73	68
Fat	4	4
kcal/gm	3.8	3.59
Casein	14	8.1
L‐Cystine	0.18	0.17
Corn Starch	49.57	10.14
Maltodextrin 10	12.5	11.63
Sucrose	10	9.31
Cellulose	5	4.09
Soybean oil	4	2.23
t‐Butylhydroquinone	0.008	0.01
Mineral Mix S10022M	3.5	3.26
Vitamin Mix V10037	1	0.93
Choline Bitartrate	0.25	0.15
Fiber (kJ/g)	0.5	1.6
C3G (μg/mg)	0	0.659

We apologize for this error.

**REFERENCE**

1.﻿﻿ Wang, Ling, Yi‐Xuan Tu, Lu Chen, Ke‐Chun Yu, Hong‐Kai Wang, Shu‐Qiao Yang, Yuan Zhang, et al. 2024. “Black Rice Diet Alleviates Colorectal Cancer Development Through Modulating Tryptophan Metabolism and Activating AHR Pathway.” *iMeta* 3, e165. https://doi.org/10.1002/imt2.165

