# Supplementary material for: Black rice diet alleviates colorectal cancer development through modulating tryptophan metabolism and activating AHR pathway
Source: Imeta. 2024 Jan 15;3(1):e165. doi: 10.1002/imt2.165 (PMC10989083; doi:10.1002/imt2.165)
Supplement: Supplementary file 1 — Figure S1: Black rice diet against intestinal tumorigenesis in AOM/DSS mouse model. Figure S2: Germ‐free mice receiving fecal microbiota transplantation exhibit altered gut microbial composition. Figure S3: The black rice diet altered gut microbial composition and increased the abundance of beneficial bacteria in AOM/DSS model. Figure S4: Black rice diet altered intestinal feces metabolite composition and enhanced tryptophan metabolism pathway in AOM/DSS. Figure S5: Black rice diet altered intestinal serum metabolite composition and enhanced tryptophan metabolism pathway in ApcMin /+ mice. Figure S6: Indole and Indole‐3‐lactic acid inhibit cell proliferation and cell junction impairment. Figure S7: The AHR pathway in the gut of germ‐free mice receiving black rice fecal microbiota transplantation was activated. Figure S8: Indole activates host AHR to inhibit colorectal cancer development. [file IMT2-3-e165-s001.docx]

**Supporting information to:**

**Black rice diet alleviates colorectal cancer development through modulating tryptophan metabolism and activating AHR pathway**

**Running title：Black rice diet antagonizes colorectal cancer development**

Ling Wang^1,2,3,4#^, Yi-Xuan Tu^1,2,3#^, Lu Chen^1#^, Ke-Chun Yu^1^, Hong-Kai Wang^1^, Shu-Qiao Yang^1^, Yuan Zhang^1^, Shuai-Jie Zhang^1^, Shuo Song^1^, Hong-Li Xu^5^, Zhu-Cheng Yin^5^, Ming-Qian Feng^1^, Jun-Qiu Yue^6^, Xiang-Hong Huang^7^, Tang Tang^8^, Shao-Zhong Wei^9^*, Xin-Jun Liang^5^*, Zhen-Xia Chen^1,2,3^*

^1^Hubei Hongshan Laboratory, Hubei Key Laboratory of Agricultural Bioinformatics, College of Life Science and Technology, College of Biomedicine and Health, Interdisciplinary Sciences Institute, Huazhong Agricultural University, Wuhan 430070, China

^2^Shenzhen Institute of Nutrition and Health, Huazhong Agricultural University, Shenzhen 518000, China

^3^Shenzhen Branch, Guangdong Laboratory for Lingnan Modern Agriculture, Genome Analysis Laboratory of the Ministry of Agriculture, Agricultural Genomics Institute at Shenzhen, Chinese Academy of Agricultural Sciences, Shenzhen 518000, China

^4^Department of Pharmaceutical Chemistry, University of California-San Francisco, San Francisco, California 94158, United States

^5^Department of Medical Oncology, Hubei Cancer Hospital, Tongji Medical College, Huazhong Agricultural University, Wuhan 430079, China

^6^Department of Pathology, Hubei Cancer Hospital, Tongji Medical College, Huazhong University of Science and Technology, Wuhan 430030, China

^7^Wuhan Myhalic Biotechnological Co., Ltd, Wuhan 430070, China

^8^Wuhan Metware Biotechnology Co., Ltd, Wuhan 430070, China

^9^Department of Gastrointestinal Oncology Surgery, Hubei Cancer Hospital, Tongji Medical College, Huazhong Agricultural University, Wuhan 430079, China

#These authors contributed equally: Ling Wang, Yi-Xuan Tu, Lu Chen.

*Correspondence: [zhenxia.chen@hzau.edu.cn](mailto:zhenxia.chen@hzau.edu.cn) (Zhen-Xia Chen), [weishaozhong@163.com](mailto:weishaozhong@163.com) (Shao-Zhong Wei), [doctorlxj@163.com](mailto:doctorlxj@163.com) (Xin-Jun Liang)

**Supplemental materials and methods**

**Test method of TM (TOF to MRM) widely targeted metabolomics**

The sample stored at -80 °C refrigerator was thawed on ice. A 400 μL solution (Methanol: Water = 7:3, V/V) containing internal standard was added into 20 mg sample, and vortexed for 3 min. The sample was sonicated in an ice bath for 10 min and vortexed for 1 min, and then placed in -20 °C for 30 min. The sample was then centrifuged at 12000 rpm for 10 min (4 °C). And the sediment was removed, then centrifuged the supernatant at 12000 rpm for 3 min (4 °C). A 200 μL aliquots of supernatant were transferred for LC-MS analysis.

T3 UPLC Conditions: The sample extracts were analyzed using an LC-ESI-MS/MS system (UPLC, ExionLC ADˈ https://sciex.com.cn/; MS, QTRAP® System, https://sciex.com/). The analytical conditions were as follows, UPLC: column, Waters ACQUITY UPLC HSS T3 C18 (1.8 μm, 2.1 mm*100 mm); column temperature, 40 °C; flow rate, 0.4 mL/min; injection volume, 2μL or 5μL; solvent system, water (0.1% formic acid): acetonitrile (0.1% formic acid); gradient program, 95:5 V/V at 0 min, 10:90 V/V at 10.0 min, 10:90 V/V at 11.0 min, 95:5 V/V at 11.1 min, 95:5 V/V at 14.0 min.

The Triple TOF mass spectrometer was used for its ability to acquire MS/MS spectra on an information dependent basis (IDA) during an LC/MS experiment. In this mode, the acquisition software (Triple TOF 6600, AB SCIEX) continuously evaluates the full scan survey MS data as it collects and triggers the acquisition of MS/MS spectra depending on preselected criteria. In each cycle, 12 precursor ions whose intensity greater than 100 were chosen for fragmentation at collision energy (CE) of 30 V (12 MS/MS events with product ion accumulation time of 50 msec each). ESI source conditions were set as following: Ion source gas 1 as 50 Psi, Ion source gas 2 as 50 Psi, Curtain gas as 25 Psi, source temperature 500 °C, Ion Spray Voltage Floating (ISVF) 5500 V or -4500 V in positive or negative modes, respectively.

LIT and triple quadrupole (QQQ) scans were acquired on a triple quadrupole-linear ion trap mass spectrometer (QTRAP), QTRAP® LC-MS/MS System, equipped with an ESI Turbo Ion-Spray interface, operating in positive and negative ion mode and controlled by Analyst 1.6.3 software (Sciex). The ESI source operation parameters were as follows: source temperature 500°C; ion spray voltage (IS) 5500 V (positive), -4500 V (negative); ion source gas I (GSI), gas II (GSII), and curtain gas (CUR) were set at 50, 50, and 25.0 psi, respectively; the collision gas (CAD) was high. Instrument tuning and mass calibration were performed with 10 and 100 μmol/L polypropylene glycol solutions in QQQ and LIT modes, respectively. A specific set of MRM transitions were monitored for each period according to the metabolites eluted within this period.

**Determination of tumor number and volume from mouse models**

The whole intestine was isolated and rinsed with ice-cold sterile PBS solution. Solid neoplastic lesions were counted for tumor number and measured for tumor volume (major diameter × minor diameter^2^/2).[1] The proximal tissue of each intestinal segment was snap frozen in liquid nitrogen and kept at −80 °C later and the distal tissue was fixed with 4% paraformaldehyde. Sections (4 mm) were stained with hematoxylin and eosin for histologic examination. Pathological types of specimens were diagnosed by two experienced pathologists who were unaware of the treatment allocation of the mice.

**Serum LPS,** **TNF-α, IL-4, IL-6, and IL-10 quantification**

Serum levels of LPS, TNF-α, IL-4, IL-6, and IL-10 were assessed using ELISA kits (LPS: catalog number RK04263, ABclonal, Wuhan, China; TNF-α: catalog number RK00027, ABclonal, Wuhan, China; IL-4: catalog number RK00036, ABclonal, Wuhan, China; IL-6: catalog number RK00008, ABclonal, Wuhan, China; IL-10: catalog number RK00016, ABclonal, Wuhan, China). All experimental procedures were conducted in accordance with the manufacturer's instructions.

**Immunohistochemistry staining**

Paraffin-embedded intestinal tissue cut into 4 μm slices by a microtome were also subjected to immunostaining for detecting the expressions of Ki-67, Claudin-3, and Occludin with primary antibodies of Ki-67 (catalog number 16667; Abcam, Cambridge, MA), ZO-1 (catalog number 33-9100; Thermo Fisher Scientific, Waltham, MA), Claudin-3 (catalog number 34-1700; Thermo Fisher Scientific, Waltham, MA), and Occludin (catalog number PA5-30230; Thermo Fisher Scientific, Waltham, MA), and 6 areas randomly selected from each section were viewed at the tumor tissue. The percentage of positive cells in each field was calculated by Image J. The specific methods are as follows:1) Correcting Image Density: (1) Click on "File" and open an image, then bring up the intensity correction window. (2) Inside the window, create a new calibration set. (3) Set the white point by setting the black level to 0 in the pop-up window. (4) Click the system button in the upper right corner, and finally click "Close" to close the window. After this adjustment, the measured optical density values will be directly proportional to the concentration. 2) Color Selection: Click on "Process," and in the segmentation section, click the dropdown menu in the upper right corner. Choose to use the HSI color system (H for hue, S for color saturation, I for intensity). Set H: 0-30, S: 0-255, I: 0-230, and then click "File" and "Save." Note that the selection ranges in HSI may need to be adjusted according to the image characteristics. 3) Setting the Analysis Environment: (1) Click "Measure," select "Count/Size," and click "Set Measurements." Generally, select IOD and the default area for measurement. The filter value for the area (default is 10) can be set to 25-50 to remove small artifacts. (2) Click "Options," and under the Contours dropdown, select "Fill." Under the Label Style dropdown, choose "None." Set smoothing to 1, and leave other options unselected. (3) In the Count/Size window, click "File" to save the environment settings. Save the environment settings file. 4) Measurement: (1) Use the irregular tool to outline the measurement area. (2) Click the "Count" button. Measurement data will appear in the View Statistics window. Read the IOD SUM value as the cumulative optical density value for this image.

**Periodic acid-Schiff (PAS) staining**

Colon sections were incubated with 1% periodic acid solution (Sigma-Aldrich) for 10 min, and with Schiff reagent (Sigma-Aldrich) for 40 min subsequently, and followed by haematoxylin dye for 5 min.

**Transmission electron microscopy**

Colon tissue samples were fixed in 2.0% glutaraldehyde in 0.1 mol/L sodium cacodylate (Electron Microscopy Sciences, Hatfield, PA) and sectioned ultrathin using a Reichert Ultracut E ultramicrotome. Tissue ultrastructure was examined with a Philips CM100 transmission electron microscope.

**Cell cycle progression**

To assess cell cycle progression, cells underwent a 24-hour serum starvation, followed by treatment in DMEM containing either 0.1% fatty acid-free bovine serum albumin (BSA)/Vehicle, Indole, Indole-3-lactic acid, CH-223191 (1 μm), Indole-3-lactic acid+CH-223191 or Indole+CH-223191 in 0.1% fatty acid-free BSA for 12 hours. Subsequently, cells were fixed in 70% ethanol and stained with propidium iodide for DNA content analysis. Flow cytometry was employed to evaluate the distribution of cells across distinct cell cycle phases.

**Bacteria Culture**

*Bacteroides uniformis* (8492) and *E. coli MG1655* (700926) were purchased from ATCC (Manassas, VA). *B. uniformis* and *E. coli MG1655* were both maintained in Anaerobe Basal Broth (catalog number CM0957; Oxoid, Hampshire, UK) at 37°C.

**Bacteria and CRC cell coculture**

CRC cells were seeded in a 96-well plate (5,000 cells/well) using Dulbecco's Modified Eagle Medium (DMEM; Gibco BRL, Grand Island, NY) supplemented with 10% fetal bovine serum. Cells were then exposed to *Bacteroides uniformis* at a multiplicity of infection (MOI) of 100 for four hours under anaerobic conditions. Following this exposure, the medium containing bacteria was replaced with DMEM supplemented with 10% fetal bovine serum, 1% penicillin-streptomycin, and 40 μg/mL gentamycin. The coculture was maintained for up to three days. Cells were subsequently trypsinized, and cell numbers were counted daily.

**Quantitative reverse-transcription PCR (qRT-PCR)**

Total RNA was extracted using the RNeasy mini kit, and cDNA reverse transcription was carried out using the TIAN Script RT Kit according to the manufacturer's instructions. The oligonucleotide primers for target genes: *Ephb2* PF: TAGCCGCCGTGGAAGAAA, PR: TGGTCCGCAGCCAGTTGTT; *Cyp1a1* PF: CCCTTACAAGTATTTGGTCGTG, PR: GAGGCTGTCTGTGATGTCCC; *Znrf3* PF: TGACCCCTTCATCTACCGC, PR: CGAGGACCCAGGAATAAACT; Esr1 PF: GGACAGGAATCAAGGTAAATGT, PR: AGGTCATAGAGGGGCACAAC; *Rspo1* PF: AAGTCAACGGTTGCCTCAA, PR: CCACTCGCTCATTTCACATT; Cyp1b1 PF: CTCTGCCGAAAAGAAAGCG, PR: CCACAACCTGGTCCAACTCA; *Ar* PF: GTTAGGGCTGGGAAGGGTCT, PR: CCGGCCTCGTTCAAAATG; *Ptgs2* PF: TGGAAAAGGTTCTTCTACGGA, PR: GCTGGTTTGGAATAGTTGCTC. The relative mRNA expression was performed using a standard ∆∆CT method to calculate fold-changes normalized to housekeeping genes for each sample.

**Western blotting**

Intestinal tumour tissue was adequately homogenized on ice in a mixture of RIPA, PMSF, and protease inhibitors. Centrifugation was spun at 13000 rpm to collect the supernatant containing total protein. Protein concentration was measured using detergent compatible protein assay (BIO-RAD, Hercules, CA). Then, 40 mg of protein was separated by 5% upper gel and 12% lower gel and transferred onto polyvinylidene difluoride membranes (GE Healthcare, Piscataway, NJ). The primary anti-β-catenin (ab32572, Abcam, 1:5000), ZO-1 (33-9100; Thermo Fisher Scientific, 1:1000), Claudin-3 (34-1700; Thermo Fisher Scientific, 1:1000), Occludin (PA5-30230; Thermo Fisher Scientific, 1:1000), PCNA (13110; Cell Signaling, 1:2000), Cyclin D1 (2922; Cell Signaling, 1:1000) were applied. Secondary antibodies were horseradish peroxidase conjugated anti-rabbit or anti-mouse. Chemiluminescence signals were detected by the ECL detection kit. The intensity of Western blotting images was determined by Image J.

**Cell culture**

Colon cancer cell lines SW620 and HCT116 were acquired from ATCC and cultured in Dulbecco's modified Eagle medium (DMEM; Gibco BRL, Grand Island, NY) supplemented with 10% fetal bovine serum (Sigma-Aldrich, St Louis, MO).

**Reference**

1. Yang, Jia, Hong Wei, Yunfei Zhou, Chun-Ho Szeto, Chuangen Li, Yufeng Lin, Olabisi O Coker, et al. 2022. “High-Fat Diet Promotes Colorectal Tumorigenesis Through Modulating Gut Microbiota and Metabolites.” *Gastroenterology* 162: 135-149.e132. <https://doi.org/10.1053/j.gastro.2021.08.041>

**Supplemental figure**


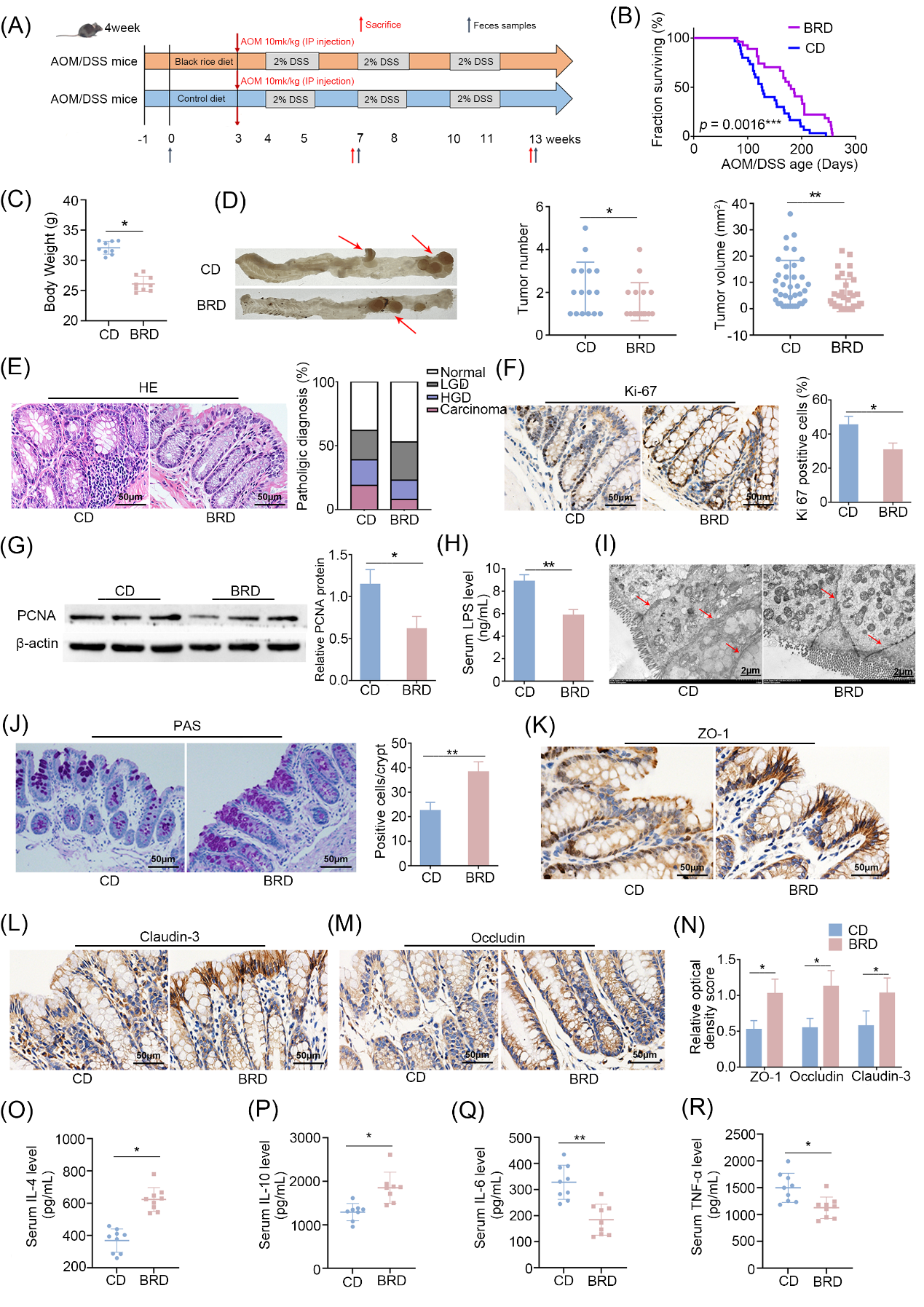


**Figure S1 Black rice diet against intestinal tumorigenesis in AOM/DSS mouse model.** (A) Experimental design for AOM/DSS CRC mouse model and WT mice subjected to either a black rice diet or a control diet. The blue arrows below the x-axis at 0, 7, and 13 represent the times when fecal samples were collected, while the bright red arrows at 7 and 13 represent the times when mouse blood and intestinal tissue samples were taken. The dark red arrows at three-week intervals indicate the times of AOM injection. (B) Enhanced survival observed in black rice diet-fed mice (n = 27 per group) compared to control diet-fed mice (n = 30 per group). (C) Body weight of black rice diet-fed and control diet-fed mice prior to sacrifice (n = 9 per group). (D) Representative colon image at the time of sacrifice. Tumor number and tumor volume in black rice diet-fed and control diet-fed mice. (E) H&E staining for pathological diagnosis of mice colons. Quantitative analysis of the pathological score employed the following criteria: 0, normal; 1, low-grade dysplasia; 2, high-grade dysplasia; and 3, carcinoma. (F) Immunohistochemical staining for Ki-67 in mice colons, accompanied by a quantitative analysis of the Ki-67 index. (G) Expression levels of PCNA protein in colon tissues of black rice diet-fed and control diet-fed mice using Western blot with quantitative analysis. (H) LPS concentration in serum of black rice diet-fed and control diet-fed mice in an AOM/DSS model. (I) Representative images of intercellular junctions captured by transmission electron microscopy. The structures highlighted by the red arrows are the focal points. (J) The number of colon goblet cells assessed by PAS staining. (K-N) IHC for the distribution of adhesion molecules ZO-1, Claudin-3, and Occludin with quantitative analysis in colon tissues of black rice diet-fed and control diet-fed mice. (O,P) Anti-inflammatory IL-4 and IL-10 concentrations, (Q,R) Pro-inflammatory TNF-α and IL-6 concentrations in serum of black rice diet-fed and control diet-fed mice in an AOM/DSS model. IHC, immunochemistry; LGD, low-grade dysplasia; HGD, high-grade dysplasia; H&E, hematoxylin and eosin. Black, black rice diet; Control, control diet. * *p* < 0.05, ** *p* < 0.01, N.S. no significant. Dot plots reflect data points from independent experiments.


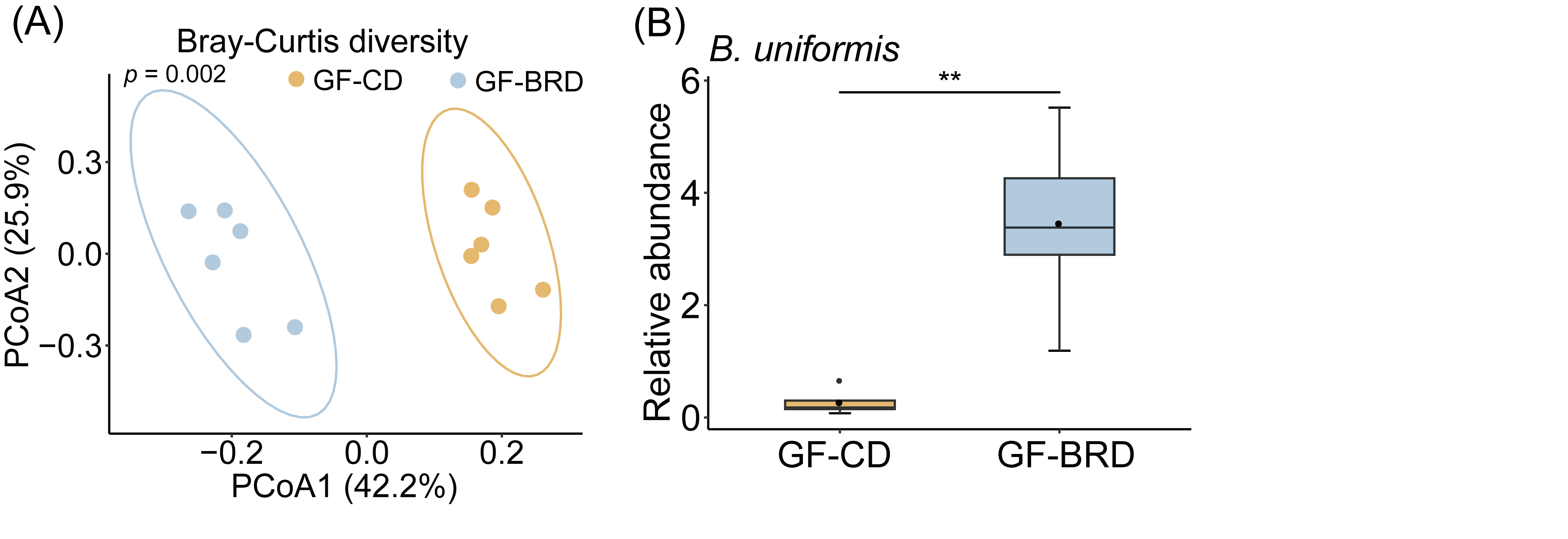


**Figure S2** **Germ-free mice receiving fecal microbiota transplantation exhibit altered gut microbial composition.** (A) Principal coordinate analysis (PCoA) analysis by bray-curtis distance (n = 6 per group). (B) The relative abundance of *Bacteroides uniformis* in GF-CD mice and GF-BRD mice*.* Data are expressed as mean ± SD. ** *p* < 0.01. Dot plots reflect data points from independent experiments.


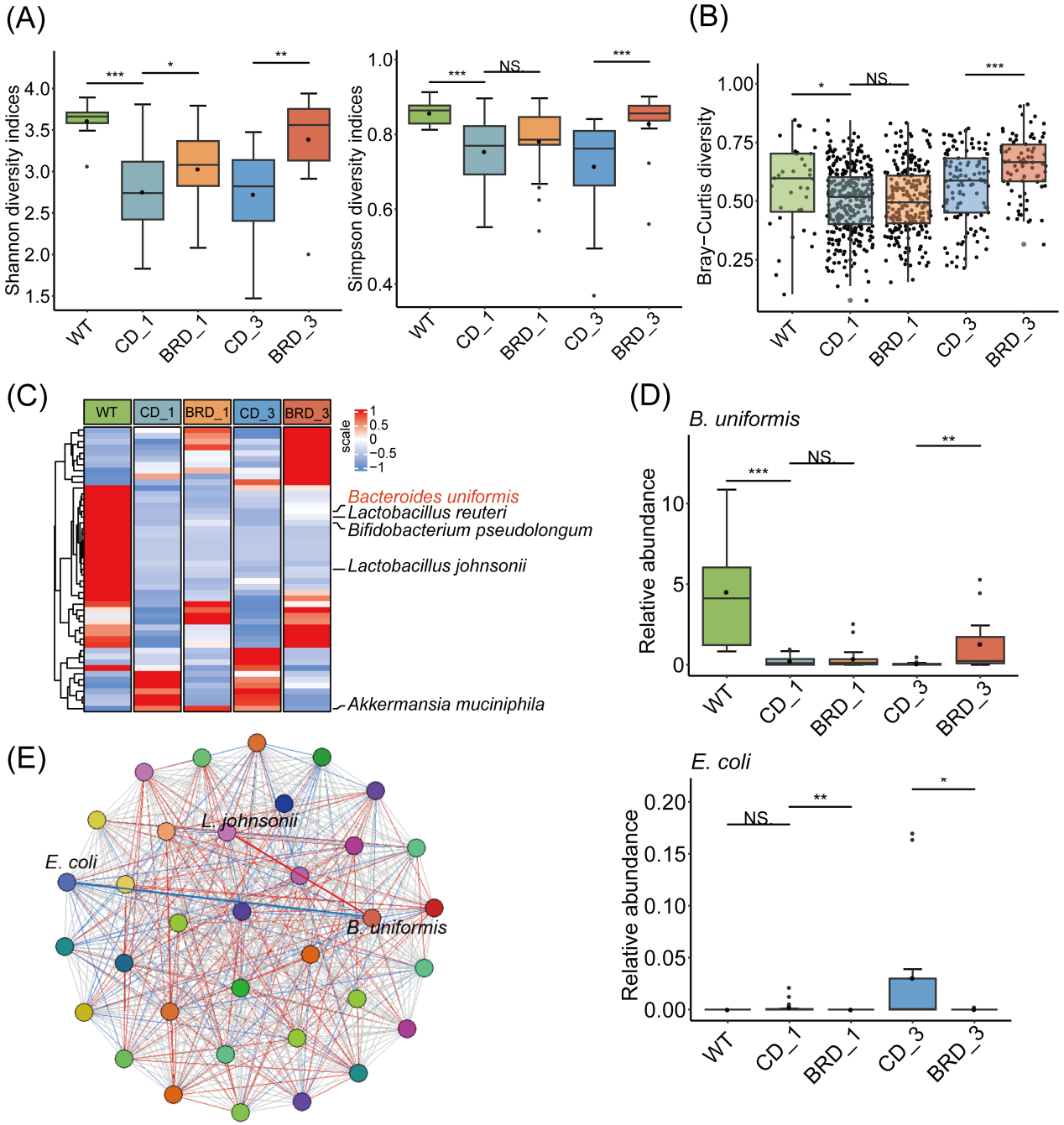


**Figure S3 The black rice diet altered gut microbial composition and increased the abundance of beneficial bacteria in AOM/DSS model.** (A) Alpha-diversity assessment using Shannon and Simpson indices for wild type mice (WT, n = 9 per group), control diet-fed mice, and black rice diet-fed mice following the first (CD_1, n = 26 per group and BRD_1, n = 23 per group) and third (CD_3, n = 16 per group and BRD_3, n = 13 per group) DSS treatments. (B) Beta-diversity analysis employing the Bray-Curtis distance metric. (C) Detection of marker microbes that differentiate black rice diet-fed mice from control diet-fed mice, as well as between wild type mice on a control diet and AOM/DSS mice on a control diet (*p* < 0.05, LDA > 2). (D) The relative abundance of *Bacteroides uniformis* and *Escherichia coli*. (E) Co-occurrence analysis: Spearman correlation coefficient between microbes. Different colors represent distinct bacterial taxa. Red lines indicate positive correlations, blue lines represent negative correlations, and gray lines denote no correlation. Data are expressed as mean ± SD. * *p* < 0.05, ** *p*< 0.01, *** *p*< 0.001, N.S. no significant. Dot plots reflect data points from independent experiments.

**
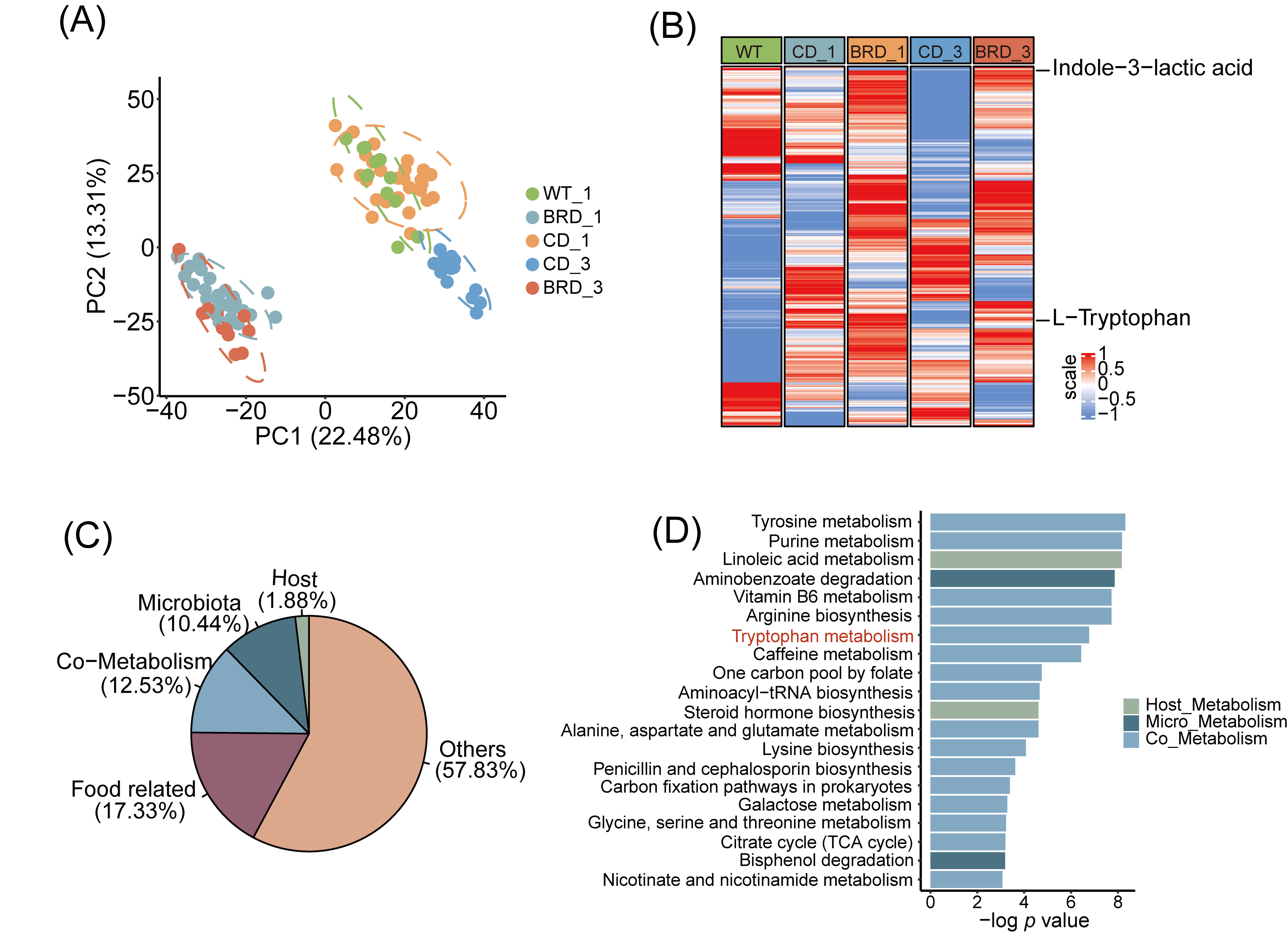
**

**Figure S4 Black rice diet altered intestinal feces metabolite composition and enhanced tryptophan metabolism pathway in AOM/DSS.** (A) Principal component analysis (PCA) plot displaying gut metabolomics profiles in wild type mice (WT_1), control diet-fed mice, and black rice diet-fed mice following the first (CD_1 and BRD_1) and third (CD_3 and BRD_3) DSS treatments. (B) Identification of marker metabolites distinguishing black rice diet-fed mice from control diet-fed mice, as well as between wild type mice fed a control diet and AOM/DSS mice fed a control diet. (C) Source attribution analysis of differential metabolites: host-derived (1.88%), microbially-derived (10.44%), shared (12.53%), food-related (17.33%), and other origins (57.83%). (D) Enrichment analysis exploring the contributions of metabolites from distinct sources. Tryptophan metabolic pathways are highlighted in red. Data are expressed as mean ± SD. Dot plots reflect data points from independent experiments.


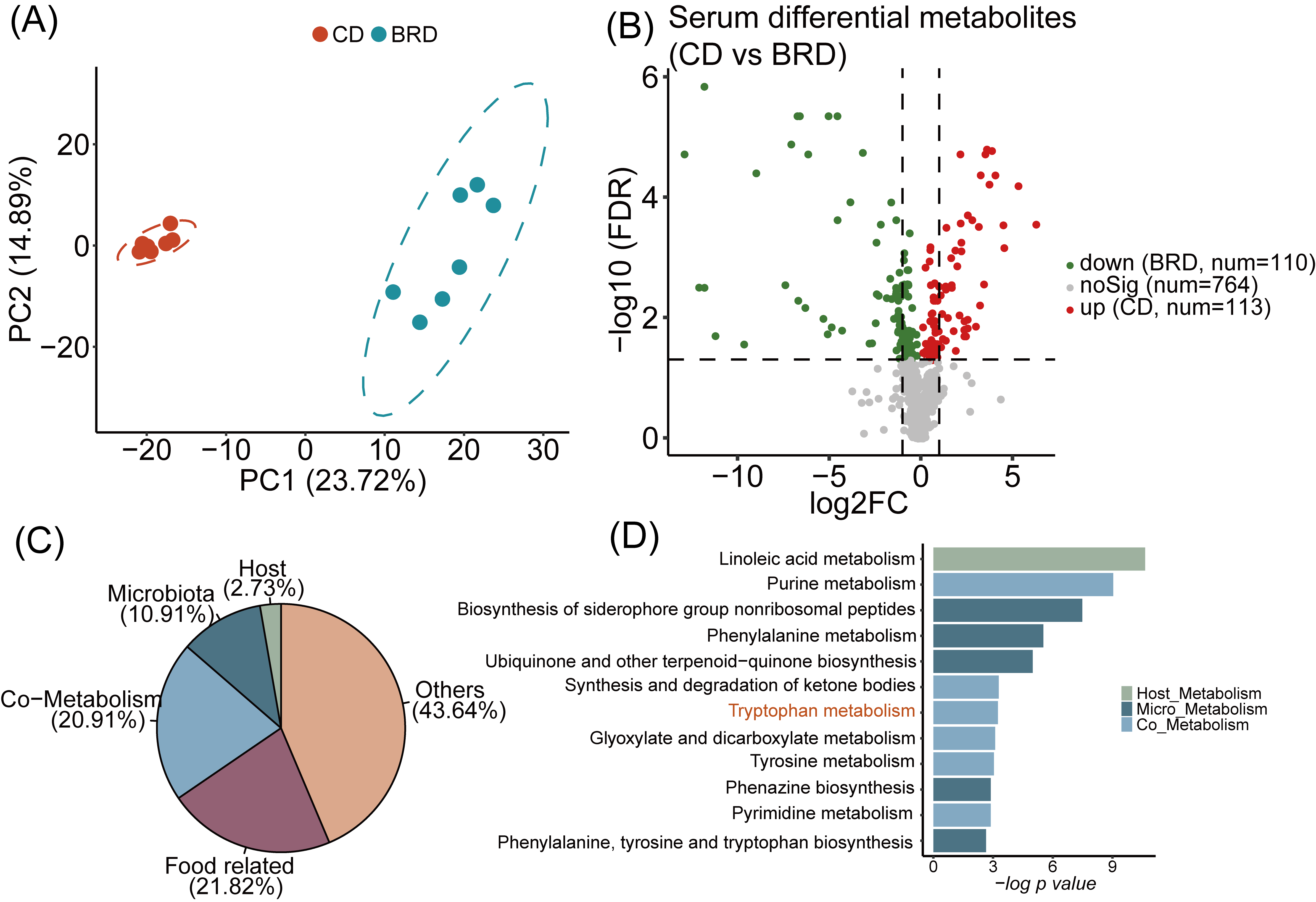


**Figure S5 Black rice diet altered intestinal serum metabolite composition and enhanced tryptophan metabolism pathway in *Apc^Min^*^/+^ mice.** (A) PCA plot was used to analyze the serum metabolomics of mice fed with control diet and black rice diet at 22 weeks. (B) Marker metabolites between black rice and control diet were screened based on differential metabolites (DEMs) with a padj-value < 0.05 and VIP > 1. A total of 223 DEMs were identified, including 110 upregulated and 113 downregulated genes in black rice diet-fed mice. (C) The analysis of the traceability of differential metabolites showed that 2.73% of the metabolites were derived from the host, 10.91% were microbial, 20.91% were common, 21.82% were food-related, and 43.64% were from other sources. (D) Enrichment analysis of metabolites from different origins was performed, and the tryptophan metabolic pathway was highlighted in red. Data are expressed as mean ± SD. Dot plots reflect data points from independent experiments.


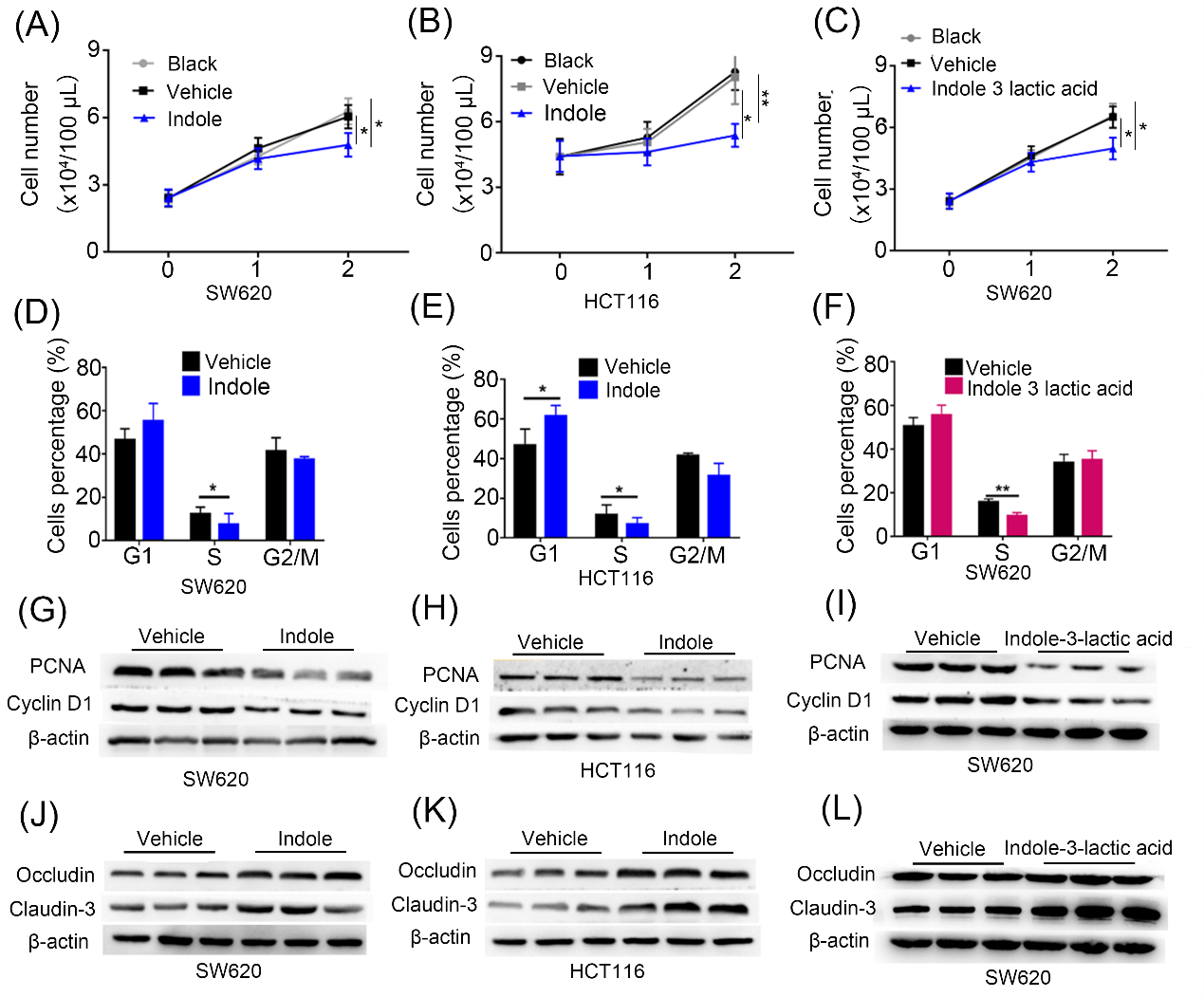


**Figure S6 Indole and** **Indole-3-lactic acid inhibit cell proliferation and cell junction impairment.** (A,B) Cell growth curves of CRC cell line HCT116 and SW620 colon cell lines treated with Indole and Vehicle. (C) Cell growth curves of CRC cell line SW620 colon cell lines treated with Indole-3-lactic acid and Vehicle (as negative control). (D,E) SW620 cells and HCT116 cells treated with or without Indole were stained with propidium iodide (PI) and analyzed using ﬂow cytometry. (F) SW620 cells treated with or without Indole-3-lactic acid were stained with PI and analyzed using ﬂow cytometry. (G,H) Expression levels of cell proliferation and cell cycle–associated proteins PCNA and Cyclin D1, in SW620 cells and HCT116 cells treated with Indole and Vehicle. (I) Expression levels of cell proliferation and cell cycle–associated proteins PCNA and Cyclin D1, in SW620 cells treated with Indole-3-lactic acid and Vehicle. (J,K) Expression levels of gut barrier function-associated proteins Occludin and Claudin-3 in SW620 cell lines and HCT116 cells treated with or without Indole. (L) Expression levels of gut barrier function-associated proteins Occludin and Claudin-3 in SW620 cell lines treated with or without Indole-3-lactic acid. The relative protein levels are normalized to those of the control β-actin. PCNA, proliferating cell nuclear antigen. Data are expressed as mean ± SD. * *p* < 0.05, ** *p*< 0.01.


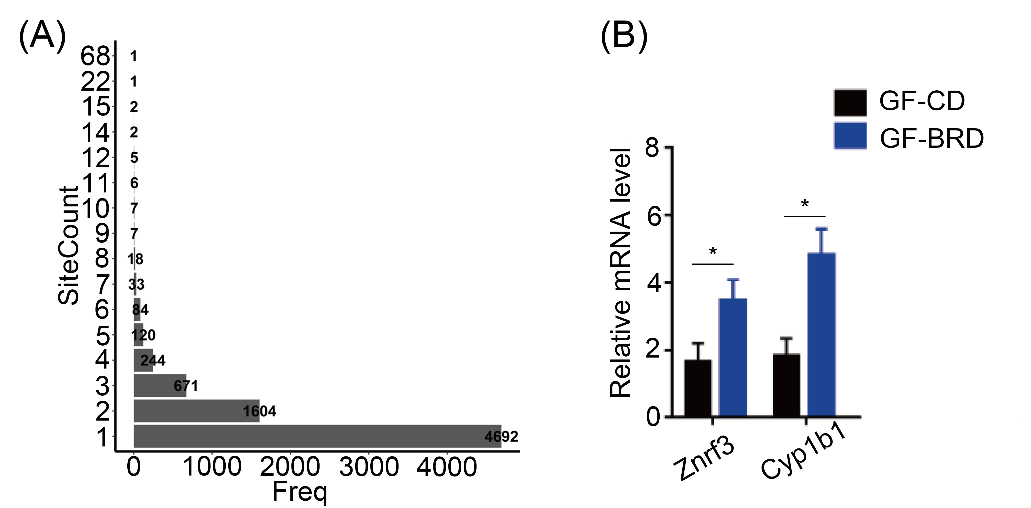


**Figure S7 The AHR pathway in the gut of germ-free mice receiving black rice fecal microbiota transplantation was activated.** (A) 7497 downstream target genes of AHR were downloaded from GTRD database, including the number of binding sites > 2 as the core target genes, there are 2805. (B) qRT-PCR results showed that black rice diet significantly promoted AHR downstream target genes expression. The relative RNA levels are normalized to those of the control β-actin. Data are expressed as mean ± SD. * *p* < 0.05. Dot plots reflect data points from independent experiments.


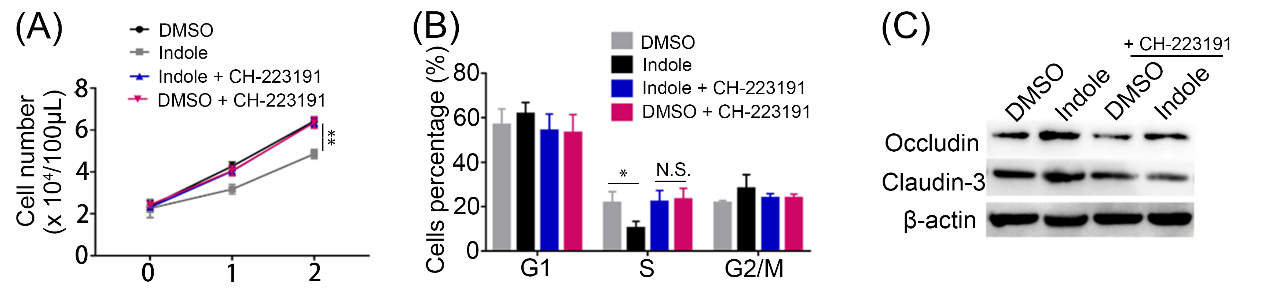


**Figure S8** **Indole activates host AHR to inhibit colorectal cancer development.** (A) Cell growth curves of CRC cell line HCT116 colon cell lines treated with DMSO, Indole, DMSO + CH-223191 (a potent and specific antagonist of aryl hydrocarbon receptor), and Indole +CH-223191. (B) HCT 116 cells treated with DMSO, Indole, DMSO + CH-223191, and Indole +CH-223191 were stained with PI and analyzed using ﬂow cytometry. (C) Expression levels of gut barrier function-associated proteins Occludin and Claudin-3 in HCT116 cell lines treated with DMSO, Indole, DMSO + CH-223191, and Indole + CH-223191. The relative protein levels are normalized to those of the control β-actin. Data are expressed as mean ± SD. * *p* < 0.05, ** *p*< 0.01, N.S. no significant.
